# A comparison of best practices for doctoral training in Europe and North America

**DOI:** 10.1002/2211-5463.12305

**Published:** 2017-09-08

**Authors:** Joey V. Barnett, Robert A. Harris, Michael J. Mulvany

**Affiliations:** ^1^ Department of Pharmacology Vanderbilt University School of Medicine Nashville Tennessee; ^2^ Department Of Clinical Neurosciences Karolinska Institutet Stockholm Sweden; ^3^ Department of Biomedicine Faculty of Health University of Aarhus Aarhus Denmark

**Keywords:** assessment, best practices, international doctoral training, mentoring, PhD training

## Abstract

The PhD degree was established in Berlin 200 years ago and has since spread across the whole world. While there is general agreement that the degree is awarded in recognition of successfully completed research training, there have been significant differences in the way doctoral training programs have developed in particular countries. There is, however, a clear global tendency to follow the programs currently used either in the United States or in Europe. To determine more clearly how US and European PhD programs are both similar and different, we have used a validated questionnaire to analyze biomedical PhD programs in four representative institutions at Vanderbilt University, University of Manitoba, Karolinska Institutet, and Graz Medical University. The analysis is based on 63 detailed questions concerning the research environment, outcomes, admission criteria, content of programs, mentoring (or supervising), the PhD thesis, assessment of the thesis, and PhD school structure. The results reveal that while there is considerable overlap in the aims and content of PhD programs, there are also considerable differences regarding the structure of PhD programs, mentoring and assessment of PhD theses. These differences are analyzed in detail in order to provide a foundation for discussion of their relative advantages and disadvantages, with a view to providing a platform for discussion of best practices. The results will be of importance in the continued development of global discussion about development of doctoral training.

**Abbreviations**
ASPIRE: Augmenting Scholar Preparation and Integration with Research‐Related Endeavors
BRET: Biomedical Research, Education, and Training
KI: Karolinska Institutet
NIGMS: National Institutes of General Medical Sciences (USA)
NIH: National Institutes of Health (USA)
ORPHEUS: Organization for PhD Education in Biomedicine and Health Sciences in the European System
TTC: time to completion

The training of clinical professionals, biomedical researchers, and preclinicians as competent researchers is essential for the continued development of medical innovations and scientific knowledge. From its earliest origins at the Humboldt University (Germany) in the 1800s, doctoral training leading to a PhD (DPhil) spread to the United States (Yale, 1865) and the UK (Oxford, 1917) and is now practiced throughout the world 1, 2. The requirement was that a PhD be awarded to younger students who had completed a prescribed course of graduate study and successfully defended a thesis/dissertation containing original research in science or the humanities. This requirement remains the basis for current PhD training in most institutions around the world. However, despite this ubiquity, there remain considerable variations in doctoral training programs between countries. It is the purpose of this paper to compare PhD programs in the United States, Canada, and Europe paying particular attention to the differences we have identified.

## Background

There have been many efforts to standardize and quality control doctoral training programs both by institutions themselves and by other organizations. In the United States, several organizations have a stake and voice in graduate education. For example, there are publications from the National Research Council of the National Academy of Sciences 3 and the National Institutes of Health (NIH) 4, the latter playing a major role in funding and reviewing both individual and institutional training programs. Other organizations such as the Association of American Medical Colleges 5, the Federation of American Societies for Experimental Biology 6, and many scientific discipline‐specific organizations also play important roles. In particular, one of these, the National Institute of General Medical Sciences (NIGMS), plays a major role in promoting and funding institutional predoctoral awards within several disciplines. NIGMS has played a critical role in shaping 7 and establishing review criteria for PhD programs that may be viewed as best practices for biomedical graduate education in the United States 8.

In Europe, particularly Continental Europe, the Salzburg documents 9, 10, 11 have provided a framework during the past decade for how PhD training should be incorporated into the overall program for higher education. Consistent with these, Organization for PhD Education in Biomedicine and Health Sciences in the European System (ORPHEUS) 12 has developed more detailed guidelines for ‘Best Practices’ within doctoral education 12, representing the cumulative opinion of medical and biomedical academic institutions in the majority of European countries, as described previously 13.

Comparing the US and European models, most differences are ones of detail, but others are more fundamental. To reveal the similarities and differences of PhD training programs in North America and Europe, we have compared proposed organizational best practices and the structure of select, representative programs. This has been conducted using the basis of a well‐validated self‐assessment document developed by ORPHEUS that is based on the ORPHEUS Best Practices document 12. In this way, we provide a solid basis for comparing and contrasting the strategies, innovations, and cultures within representative doctoral training programs conducted in two continents. We have thus identified common features of effective training programs and successful interventions that can improve PhD training.

Undergraduate university education in North America and Continental Europe has traditionally differed in its length, the former typically lasting 3–4 years (leading to a bachelor's degree), while the latter has a length of 5–6 years (often leading to a Magister or master's degree). However, in both cases, this education is the basis for admission to a PhD training program.

The majority of PhD students in the United States enter programs following the completion of a bachelor's degree. Master's degrees are not required for entry into PhD programs, although successful applicants generally have acquired significant research experience while completing their bachelor's degree. Many programs in the United States use an ‘umbrella’ entry mechanism whereby students are admitted into a common program of study built around a core curriculum that is supplemented by opportunities to sample a range of laboratories and disciplines. Students select a mentor (or supervisor in Europe) and a specific program of study. After 2 years, students conduct a qualification examination and subsequently spend 3 or more years working with a specific research project. The time for degree completion after admission is just over 6.5 years 14.

With introduction of the Bologna Process 7 in Europe, undergraduate education was split into bachelor's and master's, with the latter courses forming the basis for admission to PhD training. However, in a large number of European countries, a combined 5‐ to 6‐year bachelor's–master's system remains, with selected students transferring seamlessly to a subsequent PhD program.

Thus, although the structure of the undergraduate degree differs in the United States and Europe, the first 2 years of the US PhD correspond quite closely to the European master's degree, and the overall training experiences are similar. The Canadian PhD program is similar to European approaches, but those with medical degrees have to do 1 year of a master's course before transferring to a PhD program. In Europe, an MD gives direct access to admission to a PhD program, while in the US students still have to pass a qualifying examination before starting on their PhD research project.

## Materials and methods

The graduate programs selected for our comparison are one in the USA (Vanderbilt University School of Medicine), one in Canada (University of Manitoba), and two in Europe [Karolinska Institutet (KI) and Graz Medical University]. These institutions were chosen on the basis that they are all members of ORPHEUS and known to have typical forefront graduate programs in, respectively, USA, Canada, the Nordic countries, and western Europe. For the comparison, each institution filled in a questionnaire covering 63 detailed questions covering all major aspects of the programs. This questionnaire has been developed by ORPHEUS (www.orpheus-med.org) and validated by use in a large number of institutions. For each of the questions, the responses have been collated side‐by‐side (see Table S1) for easy comparison. In each case, we have determined whether the procedures described are (a) broadly similar, (b) differing in minor aspects, or (c) differing in significant aspects, as indicated in the table. Where significant differences have been identified, further information has been obtained from the institutions concerned.

## Results and Discussion

### Training timelines

**Figure 1:**
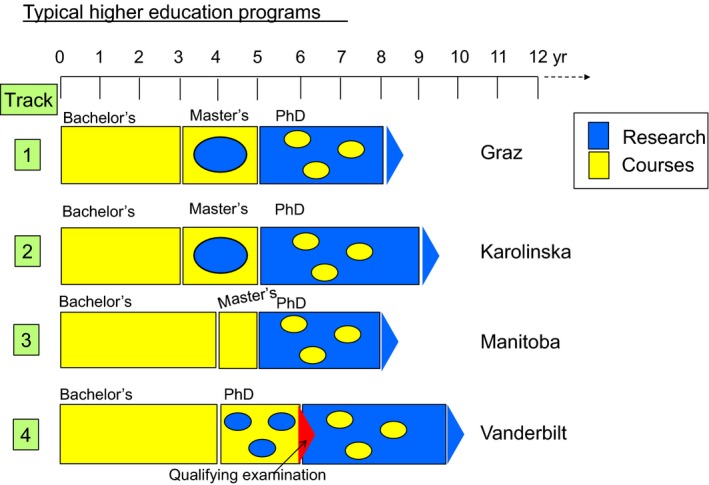
Timeline of training for nonmedical PhD students. Track 1, Graz Medical University; 2, KI; 3, University of Manitoba; 4, Vanderbilt University School of Medicine. MD trainees may have a modified timeline to the PhD degree. At Graz and KI, the MD degree gives admission to the PhD program. At Manitoba, MDs start on a master's program but transfer to PhD after 1 year. At Vanderbilt, MDs have 1 year of courses before sitting the qualifying examination.

Figure 1 compares the various training timelines (and see Table S1, line #13). The program at Graz requires for admission to the PhD program either the completion of a degree program in Medicine or Dental Medicine, or of a life science or engineer diploma/master program relevant to the dissertation topic. This is followed by a 3‐year PhD, giving a nominal 8–9 years from entering to defending a PhD thesis (Fig. 1, track 1). At KI, PhD training is set to 4 years (Fig. 1 track 2). It is a general rule at both Graz and KI that the master's or MD program has provided students with the necessary academic knowledge to start a PhD without the need for further academic courses prior to admission, and some of the master's/MD curriculum can be credited within the PhD curriculum. Courses during the PhD program are primarily related to the project, development of both specific and generic research competencies including transferable skills. Courses are typically short (5 days), occupying in total not more than about one‐sixth of the total PhD training program (Table S1, line #14).

The Canadian scheme, as exemplified by Manitoba (Fig. 1, track 3), is similar to the European scheme except that the bachelor's program is generally 4 years and a 2‐year master's. MDs have to enter a master's program, but may transfer to a PhD after 1 year of the master's. The US scheme, as exemplified by Vanderbilt (Fig. 1, track 4), is somewhat different in that transfer to PhD is direct from the bachelor's. However, the first 2 years of the PhD are primarily based on courses in much the same way as master's courses are designed in Europe. Similarly, this also includes research project rotations that often facilitate selection of a mentor. At the end of the 2 years, there is a qualifying examination (similar to the European master's examination) before being admitted to the research part of the PhD program. Thus, the initial 2 years of the Vanderbilt PhD program are very similar to the European master's program. For the Vanderbilt MD‐PhD program, MDs have 1 year of courses prior to the qualifying examination. At all four institutions, the time given to course work during the PhD is comparable.

### Time to degree

The standardized time from the start of a bachelor's to completion of a PhD varies from 8 to 9 years across these North American and European programs, but in practice may be considerably longer due to extension of the PhD program (Table S1, line #13). Students in Graz are required to include at least one published paper in their thesis and this can delay the process. KI tends to be stricter about the 4‐year limit for PhD training, but extension is possible and the average time to completion (TTC) is currently 4.5 years. Extension of PhD programs is quite common in Manitoba and this is fairly general at Vanderbilt. Reasons for extension are generally a perceived need for further time in order to complete the PhD project and to publish research papers. This perception might be that of the student but is more often that of the mentor or thesis committee. In all countries, there is a general wish, both of the institutions and the government, that PhD programs should not have prolonged extensions.

The major difference in the time frames analyzed herein is the US scheme with the direct transfer from bachelor's to PhD as opposed to the insertion of a master's between these in the other countries. The advantage of the US scheme is that the master's and PhD are integrated so that the coursework is more directed toward the subsequent research project, and placements are used to allow students the possibility to become acquainted with several research areas and mentors before defining their specific PhD project. A disadvantage is that students are locked into the scheme and should they decide that a research career is not for them they may have to leave without a degree. The other schemes thus have the advantage of more flexibility, but students might have less opportunity to make qualified choices about the topic of their PhD project and mentor.

The time limit of 3–4 years after admission in Europe has several advantages 15. These include facilitating the development of both the scope and timeline for the work to be completed, as well as planning and acquiring the necessary funding sources for the candidate and their work. Disadvantages include the difficulties encountered should unforeseen events, both those associated with the line of investigation or personal circumstances, delay the progress of the work, or force a change in experimental direction.

Recent experience in the United States has demonstrated that strict adherence to a system of regular and documented thesis committee meetings throughout the training period maintains PhD training quality while keeping time to degree after admission to 5–6 years 16. In the absence of a strict limit on the PhD TTC, this finding supports the value of the formative assessment strategy used by US programs. However, disadvantages of the lack of a specified time to degree are the potential for the student to be ‘held captive’ by unreasonable expectations of performance or research results, the lack of a specific cutoff for identifying either degree completion or dismissal, and the effects of the ever‐increasing demand for more data for a publishable research paper. At Manitoba, 4 years postadmission is the aspirational goal for TTC and is a topic of strategic focus with an emphasis on decreasing the duration. The current average TTC in the life sciences is about 5.7 years.

### Selection of PhD students

The stringency of selection procedures is naturally dependent on the number of qualified applicants and the degree to which opportunities are advertised (Table S1, line #6). At top US universities such as Vanderbilt, there are large numbers of applicants for PhD training in the biomedical sciences, and a common entry mechanism involves making formal application to a training program. Application packages commonly include a personal statement, a summary of prior research experience, academic transcripts, the results of standardized tests [most commonly the Graduate Record Examination (GRE)], and a list of recommenders who can comment on the applicant's accomplishments and abilities. Applications are evaluated by an admissions committee. Some programs or institutions may have specific requirements regarding coursework, course performance, or standardized scores, but such requirements vary greatly. Applicants may be invited to an interview during which several faculty members may meet with the applicant and successful applicants will be offered admission to the training program at the college or university. Most commonly, the admitted students are mentored by program leadership during the initial stages of their training while they consider selection of faculty mentors. Only rarely are students admitted with the intention of joining a preselected faculty laboratory.

At many European universities (e.g., Graz), PhD positions are widely advertised and selection procedures are also based on academic qualifications and research experience, with subsequent interview, somewhat similar to the US system. At other universities, both in Continental Europe (e.g., KI) and in Canada (e.g., Manitoba), although PhD positions are advertised a close relation between the student and the potential mentor has often already been established, for example, during a master's program or an MD program, and the mentor plays an important role in the selection procedure. Prior contact with the mentor can also occur in the US system, but students have to go through the normal competitive application process.

From the point of view of transparency and increasing the talent pool, the open advertising and subsequent interview procedures are clearly preferable and provide good opportunities for non‐nationals to receive PhD training. Conversely, it is commonplace that a firm prior relationship between the student and mentor is a key indicator for a successful PhD.

### Choice of PhD project

In the United States, the 2 years prior to the qualification examination allow the student time to visit different laboratories and to discuss potential projects (Table S1, line #23). The students then have time to prepare their own project and protocols. In the European and Canadian systems, the PhD students also in principle prepare their own project and protocols, and in general, these have to be approved by the institution's graduate school. In practice, there is often a logistical challenge in that students may start their programs before they have had a chance to develop their projects. Furthermore, mentors probably have strong views on what their PhD students should do, particularly if they are using their own research grants that are project specific in order to support them. In this respect, there are clear advantages for the US approach in which the 2‐year period before the qualifying examination allows the student time to develop projects as an important first process of research training.

### Financial support

At all four institutions included in our analyses, PhD students receive financial support (Table S1, line #41). This may be provided by national agencies, foundations, the institution itself, or the mentor's research grants. Such support is not universal, however, and it is the authors' experiences that in some countries students have to cover their own living expenses. Indeed, in some countries, students are also required to pay a substantial bench fee.

### Management of PhD programs and mentoring

**Figure 2:**
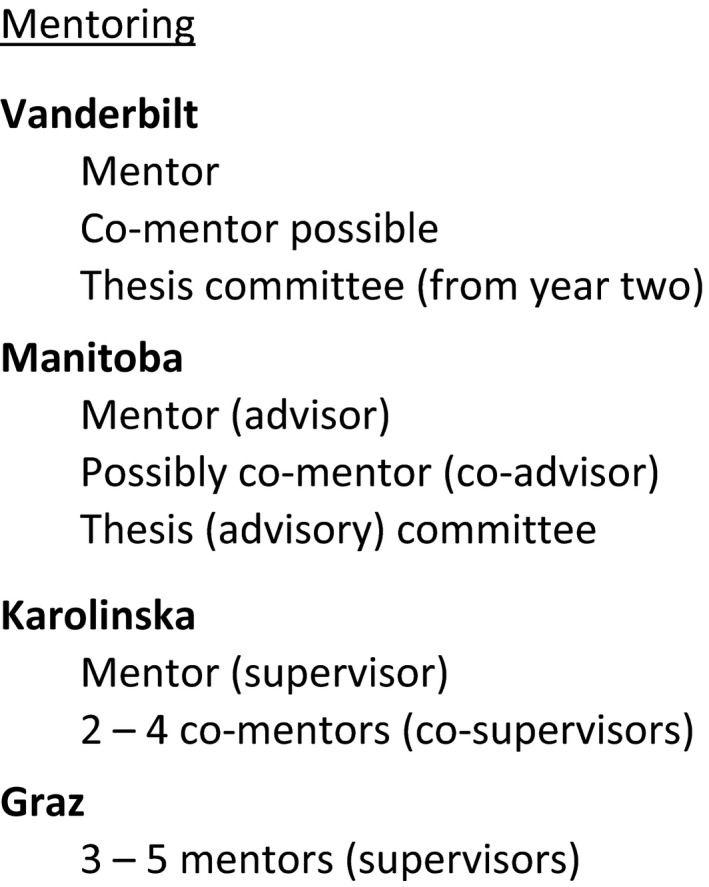
Mentoring structures. Each institution surveyed has a distinct structure for mentoring and supporting PhD trainees. Note that ‘mentors’ in the USA are called ‘supervisors’ in Europe.

In the North American model, PhD candidates have a thesis (or dissertation) committee comprising the mentor and an additional (generally 3–5) faculty members (Fig. 2 and Table S1, lines #8, #46). The panel is configured during the initial 2 years of the PhD program prior to the qualifying examination. In general, the panel meets with the student at least twice yearly, ensuring that the student receives critical feedback about both their own performance and also to permit discussion of the science being conducted. In the European model, PhD candidates are accepted into a mentor's research group and the mentor is then responsible for ensuring that the student plans and executes the thesis project during the subsequent 3–4 years. The graduate schools, to varying degrees, will monitor this process through regular annual progress reports or ‘mid‐term’ formal assessments. All of the institutions recognize the importance of professional mentorship, but only KI has compulsory mentor training courses (Table S1, lines #22, #23, #53).

### The PhD thesis

Consistent with its 200‐year‐old tradition, PhD training in most graduate schools worldwide concludes with the preparation and examination of a thesis. This will usually comprise a full review of the literature relevant to the themes in the papers, a full account of the research aims, methodological considerations, results, discussion, conclusions, and further perspectives of the PhD project. In addition, the research results will in many countries, and in all of the institutions presented herein, normally be presented as published/accepted research papers or manuscripts ready for submission. The actual number of research papers/manuscripts required is varied (Table S1, line #25). At Vanderbilt, the goal is a ‘body of work’ agreed upon by the thesis committee, the minimum generally being one accepted, first‐author paper. At Graz, at least one accepted paper is required, while KI requires at least two accepted papers. Manitoba does not have a quantitative requirement. Thus, in each of the four institutions, there is a reluctance to insist on a large number of papers, but in practice the actual number of published/accepted papers is in the order of 3, 3, 4, and 4 at Graz, KI, Manitoba, and Vanderbilt, respectively. Obviously, some students may produce a single, high impact factor paper, while others may publish several lower impact factor papers. The ORPHEUS recommendation is that the thesis should contain the equivalent of three papers, manuscripts being acceptable, and fewer papers sufficing if these are published in high impact factor journals. There is also the possibility of publishing the thesis as a monograph, although this format is less often used nowadays.

The exact content of the thesis has thus been difficult to define. On the one hand, there is general agreement that this should reflect 3–4 years of scientific work at an international level. Conversely, it is difficult in practice to quantify how this should be achieved and how to insist on a minimum level, particularly given different publishing practices in different fields. For all institutions, and for ORPHEUS, the basic principle is to allow the local assessment committee to decide.

### Assessment of doctoral training

It is inherent in a doctoral training program that there must be assessment of to what degree the training of each doctoral student has been successful. There are two general processes that are employed to this end—formative assessment in which a doctoral student's learning can be monitored and feedback given in order to facilitate improvement, and summative assessment in which doctoral student learning is evaluated according to a predefined standard. The former system is employed throughout the training period, while the latter can be employed, for example, at mid‐term and at end of the training period. At KI, the formative assessment approach has been encouraged through the introduction of intended learning outcomes (ILOs). The definition of ILOs with respect to the three domains: (a) Knowledge and Understanding; (b) Proficiency and Aptitude; and (c) Ability to Assess and Approach, for a specific research project allows for continuous appraisal of the degree of fulfillment of each ILO by the mentor(s) throughout the training program, the ambition being that there is a gradual fulfillment paralleled by increasing complexity during the program. Importantly, the same ILOs can also be included in the final summative assessment if the examiner addresses them specifically.

**Figure 3:**
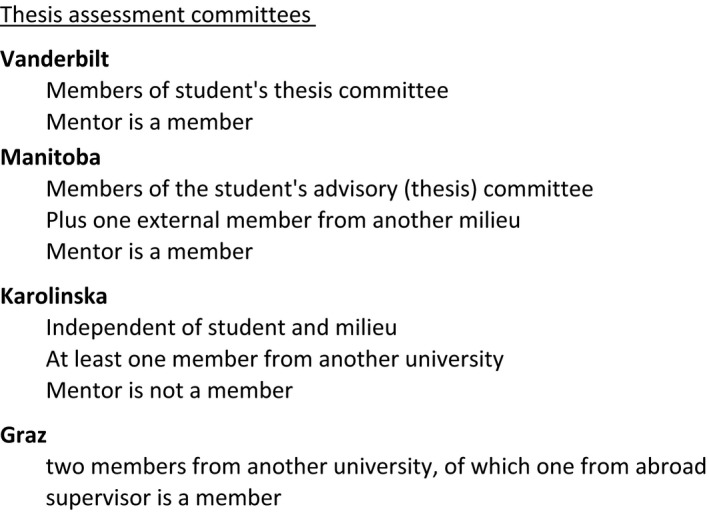
Composition of thesis assessment committees. The surveyed institutions have assessment committees that vary in size, a requirement for assessors from outside of the degree‐granting institution, and inclusion of the student's mentor (or Europe: supervisor).

In the US system, such as at Vanderbilt, assessment of the thesis is the responsibility of the doctoral student's thesis committee (Fig. 3 and Table S1, lines #17, #29, #32, #33, #60). As described above, this panel follows the student closely from enrollment and the assessment of the thesis represents a completion of the formative assessment that has occurred throughout the PhD program. The panel will not allow the student to submit the thesis unless they consider that it has achieved the necessary quality.

In the European system, the emphasis is on a final, summative assessment, the doctoral student ‘defending their thesis’ before an assessment committee involving one or more examiners. The student is awarded the PhD if the committee is convinced that the individual has demonstrated sufficient scientific knowledge, understanding of the research field, and specific methodological and contextual understanding of the studies comprising the final PhD thesis. For meaningful summative assessment, the assessment committee should be independent of the mentor and others who have contributed to the student's scientific milieu, but the degree of this independence varies between institutions. At Graz, the assessment committee traditionally consisted of professors from the university, and if necessary external professors. Until recently, the mentor was a member of the committee, thus reducing its independence. The regulations at Graz have now been changed so that at least two of the committee members should be external to the institution and one from another country; the mentor is not a member. At KI, the members of the committee must be independent of and unbiased in relation to both the doctoral student and the mentors and the project. At least one member shall come from another university. At Manitoba, the internal members have normally been members of the student's advisory (thesis) committee, but are supplemented by one external member who is independent, for example, has not collaborated or been co‐author with the mentor within the last 5 years. The Manitoba procedure is thus both formative and summative.

Within the European system, it is still implicit that there is formative assessment but, in contrast to the North American system, the responsibility for this is most often the sole responsibility of the mentor(s) which will vary in quality, and there is also variation between European institutions whether documentation of this process is required.

### Comparison of North American and European procedures for assessment

If we compare the North American and European models for assessment, there are several interesting issues to consider. The more well‐constructed and conducted formative version in North American universities ensures that the students are repeatedly made aware of both their scientific strengths and weaknesses and that they also receive detailed instruction of target areas that need work. This system should ensure that eventual challenges that occur are addressed in a reasonable time frame and it is also implicit that members of the thesis committee are active. However, it could be argued that by giving so much direct input into the student's doctoral research program the committee becomes partial and is less suited for a final formative assessment. This is one consideration why both ORPHEUS and many European institutions generally consider that an impartial examination panel should be employed during a PhD thesis defense. While the adoption of best practices such as ILO monitoring would enable European mentors to efficiently conduct formative assessments of their students, the practice is uncommon as yet and variability in the engagement and professionalism of mentors in this task leads to variability in success of the endeavor.

The impartial thesis defense that characterizes the European summative assessment conducted by seasoned experts in the specific research field should potentially be more critical than the final thesis committee assessment exemplified in the US system. However, as the expectations and understanding of what exactly should be examined during thesis defense vary widely between European institutions and indeed between individual scientists (especially between different age groups), there is a wide variation in standard of the examination. The impartiality of the examination board is best achieved by inclusion of examiners from not only outside of the student's university, but also from another country, although this requirement also varies widely and has economic implications. However, despite these good intentions, experience shows that examiners still consider it difficult to fail a student on the basis of a poor thesis defense.

The inclusion of the student's own mentor in the examination committee and the lack of a committee member from outside of the degree‐granting institution place the US system at odds with the best practices advanced by ORPHEUS. In the United States, it may be argued that the potential for the mentor to inappropriately influence the evaluation of candidates and their products (work) is tempered by the formative assessment model, during which several evaluations are performed and documented by a committee of scientists. Furthermore, the candidates are not directly accepted by and linked to the mentor as they are vetted and monitored by the graduate school and the degree‐granting program leadership. These multiple points of oversight and interest result in shared ‘ownership’ of the candidates and their work, the result being that either overly critical evaluations or the tolerance of substandard performance by a single entity, such as the mentor, is not likely to be sanctioned by other stakeholders. Clearly, for this to be successful, a culture of openness and fair evaluation must be supported by the degree‐granting institution.

We can conclude that the European model primarily relies on summative assessment of the candidate's work, while the US model primarily uses formative assessment. In summative assessment, the focus is on the outcome or end product of the candidate, whereas formative assessment evaluates and summarizes the candidate's performance over time. Both processes are certainly valid as the product must meet a specific standard in the discipline with regard to rigor, original work, and impact, while repeated evaluation over time provides an opportunity for the candidate to receive feedback as they encounter intermediate goals and challenges during the course of completing a thesis. The worst‐case scenarios would be a student in the US system that receives too much scientific input from their thesis committee at the expense of their own development of critical scientific thinking, and an unworthy student in the European system being granted their examination by an uncritical assessment committee. Presumably, the ultimate best practice would encompass structured formative assessment at defined time periods during the doctoral program, as well as an impartial final summative assessment. Importantly, this illustrates that academic institutions on both sides of the Atlantic Ocean could learn elements of best practices of doctoral education from each other that could be incorporated into their own future best practices.

### Career development

With the increasing realization that only a minority of PhD graduates will continue to use their talents within academia 15, the need to ensure that PhD training provides competences for a wide range of career options has increased during recent years. Of the four institutions considered herein, Vanderbilt has the most comprehensive career development program (Augmenting Scholar Preparation and Integration with Research‐Related Endeavors, ASPIRE) that is funded by a recent NIH initiative 17 (Table S1, line #51). This program has considerable resources and aims to empower and prepare biomedical sciences PhD students and postdoctoral scholars to make well‐informed career decisions. Career information is provided throughout training and students complete individual development plans annually. This instrument includes a mentor and student assessment of progress and goals that must be discussed by both parties. Methods include department‐ or program‐run events to meet with alumni and Biomedical Research, Education, and Training (BRET) office career symposia. Program directors and faculty provide students with advice. Forms that review topics covered at each committee meeting include whether career discussions occur. ASPIRE provides resources and support to trainees to broaden their experiences and help them transition efficiently to research and research‐related careers in both academic and nonacademic spheres. Specifically, ASPIRE offers professional development workshops, career exploration opportunities, and training enhancement.

At Manitoba, career workshops are held. Specific career development packages are not held at Graz, but a wide range of courses in transferable skills is available. In addition to these, KI has a specific central career service that organizes ‘career days’ activities such as the possibility of meeting representatives of the industrial biomedicine sector.

Given constraints on time and resources, it may be discussed how much effort should be put into career development, or whether it should be accepted that completion of a PhD program in itself provides the students with the competences that are needed to obtain employment both within and outside of academia. However, unless PhD training is perceived by students to be relevant with respect to available opportunities, there is a danger that the PhD will become less attractive, even to the brightest students.

## Conclusions

‘Best practice’ in doctoral training is not a ‘one‐size‐fits‐all’ universal concept, and we consider that flexibility is thus imperative. There are certain aspects that are globally applicable to all international educational institutions, some that are pertinent at a national level and a few aspects that might be relevant locally. However, the latter category should not result in the perpetuation of historical traditions without modern practical applicability. The processes of self‐reflection and self‐evaluation that the ORPHEUS Best Practice tool facilitates is one example of how educational institutions can identify their own areas of strengths and weaknesses, and indicate areas for improvement. By comparing and contrasting practices between institutions, and between continents as we have done here, we are able to broaden our perspectives and learn from each other. Considering the ever‐increasing globalization of employment within the science sector, so that postdoc periods are often conducted in a different country, it is of additional benefit for knowledge or at least appreciation of different PhD training systems on different sides of the globe. The unifying factor in educational institutions throughout the world should be the ambition to increase quality assurance in doctoral education in order to provide society with new generations of fully trained and inspired professionals.

## Author contributions

MM conceived of the project; MM, RH, and JVB designed the project; MM, RH, and JVB acquired the data; MM, RH, and JVB analyzed and interpreted the data; MM, RH, and JVB wrote the manuscript.

## Supporting information


**Table S1**. Responses from the participating institutions (Vanderbilt University School of Medicine, University of Manitoba, KI, Graz Medical University) to the ORPHEUS questionnaire.
