# Supplementary material for: A comparison of best practices for doctoral training in Europe and North America
Source: FEBS Open Bio. 2017 Sep 8;7(10):1444–52. doi: 10.1002/2211-5463.12305 (PMC5623696; doi:10.1002/2211-5463.12305)
Supplement: Supplementary file 1 — Table S1. Responses from the participating institutions (Vanderbilt University School of Medicine, University of Manitoba, KI, Graz Medical University) to the ORPHEUS questionnaire. [file FEB4-7-1444-s001.pdf]

Supplementary Table 1

Responses from the participating institutions (Vanderbilt University School of Medicine, University of Manitoba, Karolinska Institutet, Graz Medical University) to the ORPHEUS questionnaire. The column on the right shows the extent of similarity between the institutions for each of the areas indicated (1, broad agreement; 2, minor differences; 3, significant differences). The data were collected 2013-2015.

Line #	Brief description (with ORPHEUS reference to 2012 edition of Best Practices document)	Vanderbilt	Manitoba	Karolinska	Graz	Comparison: 1, broad agreement; 2, minor differences; 3, significant differences
**Basic Standards**						
**1. Research environment**						
1	*There should be a strong research environment around every PhD project, either within the institution or within collaborating institutions. (BS1.1)*	Vanderbilt University Medical Center has built a strong reputation as a leader in medical education, research and patient care throughout the Southeast and the nation over the course of its 136-year history. At its heart the Medical Center is driven by discovery and the immediate incorporation of new knowledge into innovation in patient care and physician and nurse education.	This standard is met. The Faculty of Health Sciences has a strong record in research productivity and the role of graduate students is prominent in that success. Our documentation shows strong support of research through facilities, platforms, collaborations and strategic plan. The research environment for PhD projects is a critical criterion for the approval of a PhD student/research program.<br><br>*http://umanitoba.ca/faculties/health_sciences/medicine/research/bscp.html*<br>*http://umanitoba.ca/faculties/health_sciences/medicine/research/regen_med.html*<br>*http://www.triumf.ca (The U of M is a full partner in*	There are 22 Departments within KI encompassing the following research areas: Neuroscience, Infection Biology, Inflammation, Cell and Molecular Biology, Physiology & Pharmacology, Clinical Medicine (all specialisations e.g. surgery, dentistry), , Public Health, Healthcare sciences, Biosciences, Laboratory Medicine, Epidemiology & Biostatistics, , Developmental biology, Environmental Medicine, Medical Pedagogics.<br>See www.ki.se (pull-down menu to the right-hand-side) for more information about specific Departments.<br>See http://ki.se/ki/jsp/polo	Research at the Medical University of Graz (MUG) is focused on four main areas (cancer research, cardiovascular research, molecular based lipid-associated disorders and neuroscience) and an interdisciplinary theme (sustainable health).<br>The members of the PhD faculty are active in all research fields and interdisciplinary topics. Thus, the candidates and their projects are integrated in terms of content and collaboration into the profile-defining research networks of MUG. Due to organizational reasons, PhD candidates are assigned to the institutes and clinics of their respective supervisors and work as research	1

			TRIUMF)<br>*http://www.lightsource.ca*<br>*(The U of M has an active interaction with Lightsource)*	poly.jsp?l=en&d=135 for more information about research at KI.<br>Quality of the whole research environment for the doctoral candidate is assessed by the Departmental Admissions Board at the time of application for PhD studies.	associates in their work groups. Thus, they are also embedded in the institutional, scientific and social context of the organizational units.	
2	*Facilities should be compatible with the requirements of completing the PhD project. (BS1.2)*	Biomedical research at Vanderbilt has long been recognized for its contributions to the advancement of medicine. The School of Medicine claims two Nobel Laureates, Earl W. Sutherland Jr., in 1971, for his discovery of the metabolic regulating compound cyclic AMP, and Stanley Cohen, in 1986, for his discovery (with a colleague) of epidermal growth factor. The Medical School's reputation for outstanding research is reflected in the amount of federal and private support it receives. In the most current ranking (2013), Vanderbilt ranked 9th in NIH grant support having received $292,413,440 in competitive funds. nts. Major translational research initiatives at Vanderbilt are moving discoveries from the bench to the bedside and will transform health care and health care delivery.	The research facilities in the College of Medicine and our collaborating institutions are internationally competitive, as required for the national/international sources of our research funding. Beyond the research facilities that our individual research groups have built, we have invested strongly in core facilities (platforms) to promote the collaborative, college-wide interactions required for research and training.<br><br>*http://umanitoba.ca/faculties/health_sciences/medicine/research/bscp.html*<br>*http://www.triumf.ca*<br>*http://www.lightsource.ca*	Whenever a PhD student applies for admission the working environment, including the psychosocial environment, and the physical space and facilities therein or available to the project, are judged by the admissions committee. This not only encompasses lab spaces with relevant technical apparatus but also office facilities, including computers. This may also include economic provision for travel to an international laboratory within a scientific collaboration for the purpose of using a specific facility.	A large variety of state-of-the-art instrumentations and facilities are available to the research groups, which provide the candidates with an excellent base to pursue their thesis projects. The Rectorate of MUG warrants that access to all facilities and lab equipment is granted to all doctoral candidates. In addition to standard laboratory equipment for individual projects, a comprehensive set of specialized infrastructure and core facilities is available. For more information see: http://zmf.medunigraz.at/en/core-facilities/<br>http://www.medunigraz.at/fileadmin/forschen/files/folder_research.pdf<br>Moreover, Biobank Graz provides one of the world's largest collections of tissue and blood samples: http://www.medunigraz.at/en/research/organisation-and-services/biobank-graz/	1
3	*Research consistent with*	Vanderbilt adheres to all	This standard is met. All	Control of the necessity for	At MUG, an ethics	1

	international ethical standards. (BS1.3)	federal and state standards regarding human and animal subjects. Standing committees for evaluating human and animal subjects are in place with proper certification for clinical facilities and animal facilities. Vanderbilt meets or exceeds all standards for training in the Responsible Conduct of Research.	research is vetted for human ethics and animal ethics as required. In addition, all graduate students are required to take an on-line course in academic integrity.<br><br>http://umanitoba.ca/faculties/health_sciences/medicine/ethics/index.html<br>http://umanitoba.ca/research/orec/ethics/animalcare_committees.html<br>http://umanitoba.ca/faculties/graduate_studies/registration/grad7500FAQ.html	ethical permits for a given doctoral education project is made (1) at admission by the Departmental admissions board, (2) at halftime review by the review panel, and (3) upon application to defend the thesis by the KI dissertation committee. A taught course in ethics (corresponding to 1.5 ECTS) is a compulsory training requirement for all doctoral students and must be completed within the first 2 years of study (see 'General Syllabus for Doctoral Students' page4).	commission is established. The commission appraises clinical trials of drugs and medical products, the application of new medical methods and applied medical research involving human subjects in accordance with the principles of the Declaration of Helsinki, the ICH-GCP guidelines and in compliance with the relevant provisions of the Austrian Medicines Act, the Austrian Medical Devices Act, the Styrian Hospitals Act, the Federal Law on hospitals and convalescent homes, and all other relevant legislation for ethical clearance: http://www.medunigraz.at/en/research/organisation-and-services/ethics-committee/ Whenever applicable, ethical permissions and animal experiment licenses are applied for.	
**2. Outcomes**						
4.	*PhD programmes should provide PhD candidates with competences to become qualified and independent researchers, according to principles of good research practice. (BS2.1)*	Students who complete a year of interdisciplinary training, join one of the 10 departments or programs (Biochemistry, Biological Sciences, Cancer Biology, Cell and Developmental Biology, Human Genetics, Microbiology and Immunology, Molecular Physiology and Biophysics, Pathology, Pharmacology, and Neuroscience). Training	Theoretical training – Courses – all programs have approved minimum course requirements to ensure that students are competent regarding knowledge in their fields.<br>Practical training – a prominent research component, supervised by a well-funded Faculty member and evaluated by a local committee and an	Developing as an independent researcher is a basic Intended Learning Outcome (ILO) defined by the Swedish Higher Education Ordinance (see 'Rules for Doctoral Education' page 6), and is incorporated into every PhD student's Individual study plan. Evaluation of progress to achievement of this aim is made at both	Transferable skills are strongly anchored in the study program. The degree program also imparts the ability to act responsibly, especially with regard to the impact of technological change in biomedical research, health care and society (biomedical ethics, good laboratory and clinical practice, animal rights). The project work of the PhD	1

		begins with mentor selection where students are guided by senior faculty and program directors. Careful mentoring by a team of faculty monitor student progress throughout the programs. Insistence on presentation of research, publication, and demonstration of independent thought are common features of each program.<br>Communication skills, written and oral, are emphasized within each training program. Grant writing skill development underlie the written portion of qualifying examinations.<br>Competencies are evaluated in several ways. These include formal communication courses where students are evaluated by defined assessment bench marks, individual examination by a committee of faculty mentors occurs for qualifying examination, journal clubs and works in progress may include peer assessment or faculty assessment, and the thesis committee evaluates communication, independence, and scientific productivity in meetings that occur at least every 9 months.	external expert as being at a level with their peers nationally/internationally.<br>Communication skills: Journal clubs, departmental seminars, "3-Minute Thesis", EAL courses as appropriate, and specific courses in teaching (with formal evaluation) are offered.<br>Grant-writing courses, with proficiency requirements for independent critical thought, are offered.<br>Students are reviewed for progress by their advisory committees regularly (at least annually) and the results reported to FGS.<br>As per the websites listed below, there are numerous resources for our students in these areas.<br><br>http://umanitoba.ca/student/bannatyne/workshops.html<br>http://umanitoba.ca/student/academiclearning/<br>http://umanitoba.ca/student/academiclearning/workshops/index.html<br>http://umanitoba.ca/student/academiclearning/writing_support/online_tutor.html<br>http://umanitoba.ca/student/academiclearning/grad_resources/index.html	halftime review and during thesis defence by dissertation committees. An annual Departmental review allows for annual modifications of individual study plans, a primary cause being incorporation of new projects designed partly/wholly by the students themselves, indicating some degree of scientific independence. PhD students have the possibility of taking part in various activities offered by the KI Careers service, the Doctoral Programmes, by the Board of Doctoral Education or by Departments themselves, including specific training of leadership, scientific commercialisation/patenting, pedagogic and teaching skills. Through these activities in combination with training in the generic skills such as scientific presentation, statistics, scientific writing, philosophy of science and research ethics, many of the attributes expected of an independent researcher can be developed.	candidates in the teams of their supervisors makes them familiar with the following general skills: ability to work effectively in a team, ability to analyse information, ability to communicate effectively, ability to learn independently and to obtain scientific knowledge, awareness of ethical and professional responsibility, leadership skills. In addition, summer schools and workshops provide hands-on training in presentation skills and scientific writing. These acquired skills are put to work in retreats, thesis seminars and international conferences.	
5	*A PhD degree should also be of benefit in a career*	Career information is provided throughout	The following resources are	Most of the skill sets described in BS2.1 are of	1) The PhD program offers candidates several courses	2

	outside academic or clinical research (problem solving, analysis, evaluation, technology transfer etc.). (BS2.2)	training.<br><br>All trainees are required to complete Individual Development Plans annually. This instrument includes a mentor and students assessment of progress and goals that must be discussed by both parties. Methods include department or program run events to meet with alumni and BRET office career symposia. Program directors and faculty provide students advice. Forms that review topics covered at each committee meeting include whether career discussions occur.<br><br>A recently funded NIH initiative, the Vanderbilt ASPIRE program, is focused on career development.<br><br>ASPIRE provides resources and support to trainees to broaden their experiences and help them transition efficiently to research and research-related careers in both academic and nonacademic venues. Specifically, ASPIRE offers professional development workshops, career exploration opportunities and training enhancement. https://medschool.vanderbilt.edu/aspir	offered:<br>Careers workshops – hosted by our Graduate Students Association and by the Office of Student Services<br>Seminars are presented by our Office of Technology Transfer<br>TIPS and CHET courses (Teaching Improvement)<br>These are offered throughout the year.<br>http://umanitoba.ca/student/bannatyne/workshops.html<br>http://umanitoba.ca/research/tto/index.html<br>http://intranet.umanitoba.ca/academic_support/catl/programs/chet.html<br>http://intranet.umanitoba.ca/academic_support/catl/workshops/243.html	use in careers within the pharmaceutical industry, within clinical posts, within non-scientific appointments in society (e.g. as bank analysts, governmental investigators).<br>In a recent investigation of graduated alumni 88% stated that use of research skills were part of their current employment.	related to general principles and skills, e.g. biostatistics, bioethics, presentation skills.<br>2) The PhD program organizes journal clubs where the candidates discuss relevant papers with their PIs.<br>3) The PhD program organizes thesis seminars in which PhD candidates present their work before their fellow candidates and discuss the progress of their work. In this endeavor they practice to be part of the scientific community.<br>4) Doctoral Day: Every year MUG organizes a Doctoral Day where candidates present their work to a larger audience. The best presentations are awarded with a prize. This gives the PhD candidates the opportunity to exercise their presentation and communication skills.<br>5) Summer Schools: MUG organizes Summer Schools for PhD candidates each year. In these schools the candidates are trained in scientific writing and self-presentation in interview situations, and made familiar with issues of broad implications such as bioethics and animal rights.	

		e/ or				
3. Admission policy and criteria						
6.	*PhD candidates should be selected on the basis of a competitive and transparent process. (BS3.1)*	The Interdisciplinary Graduate Program (IGP) was initiated in 1992 to coordinate the recruitment and first year core curriculum of graduate students in the basic biomedical and biological sciences in the School of Medicine and the College of Arts and Science. The IGP has served as a model for similar programs across the country. Beginning in November, the IGP admissions committee, consisting of 1 faculty member from each participating program or department, decides who to invite for an interview. Prospective students are hosted at recruiting visits over a 10-12 week period in the spring; successful applicants matriculate in the fall. MD,PhD students have a separate admissions process. For IGP, for the 70 or so admitted, approximately 300 students are interviewed during campus visits.	Prospective students apply to the FGS for admission Applicants consult with their desired graduate program(s) regarding availability of supervisors; upon an appropriate match being achieved (student/supervisor, re: academic suitability, funding, supervision available), the application is vetted by the departmental graduate program committee and their recommendation for acceptance is transmitted to FGS. FGS vets academic qualifications (if the previous academic record is from another institution) and communicates acceptance to the applicant. *http://umanitoba.ca/faculties/graduate_studies/admissions/min_requirements.html* http://umanitoba.ca/faculties/graduate_studies/media/cte_selection_report.pdf http://umanitoba.ca/faculties/graduate_studies/media/PhDProgramOfStudy_v5.pdf	From January 2014 onwards all doctoral positions at KI have to be advertised and selection made in competition.<br>In accordance with the Swedish Higher Education Ordinance, exceptions include:<br>when admitting a doctoral student who is to complete the course or study programme within the framework of employment by an employer other than the higher education institution<br>when admitting a doctoral student who has previously begun doctoral studies at another higher education institution, or (3) if there are similar special grounds.	Following an international call, PhD candidates are selected by a three-step procedure. First, written applications are ranked by the faculty of principal investigators (PIs) following skype interviews with the preselected candidates. Second, the top candidates are invited to a personal hearing in Graz, the expenses being covered by MUG. The hearing involves a formal presentation of the candidates' scientific work experience, an interview by members of the faculty, informal talks with preferred PIs and visits to the labs of interest. Third, the final selection of the PhD candidates is taken by a joint decision of the faculty of PIs. Since the PhD program is conducted in English, the selection process is also conducted in English.<br>The selection procedure which is administered by the Dean and Office for Doctoral Studies is scheduled such that admission is possible at the start of each semester (October/March), taking into account the timeline of the formal employment processes involved.	1

7	*Applicants for PhD programmes should have an educational level corresponding to a master's degree. (BS3.2)*	Most students enter IGP, QCB, and MD,PhD training with a 4-year Bachelor's degree. A small percentage of student hold a Master's degree. The PharmD, PhD program selects students who are current PharmD students. MD-PhD students enter a fast-track course (1 year) leading to the qualifying exam.	A MSc or equivalent is required.<br>Applicants holding an MD degree are required to be admitted to a Masters program and after one year may be considered for admission to the PhD program.<br>We also offer an MD/PhD program which accepts medical students to a 7+ year integrated program whose requirements satisfy the requirements of both degrees.<br>http://umanitoba.ca/fckeditor/editor/faculties/health_sciences/medicine/research/grad_undergrad/6696.html	A person meets the general entry requirements for doctoral education if he or she:<br>has been awarded a degree at advanced (second-cycle) level has satisfied the requirements for courses comprising at least 240 credits of which at least 60 credits were awarded at advanced level, or<br>has acquired substantially equivalent knowledge in some other way in Sweden or abroad.<br><br>Generally a master's degree is a *de facto* requirement.<br><br>The higher education institution may permit an exemption from the general entry requirements for an individual applicant, if there are special grounds.<br><br>http://ki.se/ki/jsp/polopoly.jsp?l=en&d=29411&a=29596	Required for admission to the PhD program is either the completion of a degree program in Medicine or Dental Medicine, or of a life science or engineer diploma/Master program relevant to the dissertation topic. Admission may also be granted if an equivalent degree program has been completed at a recognized Austrian or foreign tertiary educational institution. In order to successfully complete the PhD program, candidates are required to possess both the relevant knowledge in the scientific field of the thesis project and sufficient English language skills. Applicants for the PhD program should be able to demonstrate superior academic performance, and their previous academic career should demonstrate an exceptional aptitude and motivation for scientific work.	1
8	*Before enrolment or at clearly defined times during the programme, the institution should evaluate and approve:*<br>*- Scientific quality of the project*<br>*- The likelihood that the project can result in a thesis of the required standard within the timeframe*	Ph.D. projects in the participating programs is approved at the time of the qualifying examination. In general, a portion of the qualifying examination includes the presentation of a written proposal that is defended to a faculty committee. In most cases this is the student's dissertation committee.	Only supervisors who have been approved by the Faculty of Graduate Studies (PhD or equivalent, mentoring experience and having an appointment with a unit that has a graduate program) may supervise PhD students. MDs may, as approved on a case-by-case basis by FGS (active researchers, experienced	Admission of doctoral students is delegated to the Head of Department where the doctoral student will be registered. Each department has a Director of Doctoral Education who with a Departmental Admissions Board (comprising representative researchers and students) is responsible for preparing	*Scientific quality of the project:* Most PhD projects are funded by peer-reviewed grants. Hence they have been subjected to competitive assessment by external reviewers. PhD projects funded directly by MUG are not externally assessed but given only to faculty members with an excellent track record in	1

	- The possibility for the PhD candidate to provide creative input during the project<br>*- Qualifications of the nominated supervisors. (BS3.3)*	Occasionally, this presentation may occur just after the successful completion of the qualifying examinations as a separate evaluation. The proposal is a cooperative venture written by the student with input from the mentor. Quality and feasibility is evaluated.<br><br>The dissertation committee will monitor student progress by meeting at intervals no greater than 9 months. The committee includes 4 faculty within the Ph.D. granting unit and one outside member. Additional members may be appointed for specific expertise. All are appointed by the graduate school.	mentors, members of graduate departments), be accepted as PhD supervisors.<br>The PhD project is approved, upon presentation by the student, to the advisory committee. At least annually, with a deadline provided by the FGS, the student and advisory committee meet to review progress and make recommendations.<br><br>http://umanitoba.ca/faculties/graduate_studies/media/Progress_Report_2015.pdf<br>http://umanitoba.ca/faculties/graduate_studies/media/Phd_Thesis_Proposal.pdf	cases, organising and evaluating the admissions seminar, as well as submitting a proposal for a decision on admissions that serves as a guide for the Head of Department. Aside from the general and specific entry requirements, the individual study plan, funding plan, organisation and quality of supervision and of the research environment are all assessed for each case, and only those which are considered to promote doctoral education of the highest quality are recommended for admission.<br>An annual review is submitted to and reviewed by the Departmental Director of Doctoral Education, in which progress in achievement of intended learning outcomes, supervision, funding and scientific progress is assessed. Poor outcomes stimulate discussions with the Director, supervisors and students in order to strive for improvement.<br>A halftime review is conducted after 2-years of full-time equivalent study time. After proposal by the supervisor, the Head of Department will appoint a review committee	publication and external funding. In addition, the quality of the projects is assessed by the spokesperson of the PhD program, the Dean of Doctoral Studies and the Dissertation Committee.<br>*Likelihood of a successful thesis project:* The progress in the work on the thesis project is continually overseen by the supervisor. The candidates present and defend the thesis projects including milestones and time plans in front of the faculty before the end of the first semester. Every year the candidates present the progress in their projects before the faculty as well as before their Dissertation Committee. Furthermore, PhD candidates are required to submit a progress report in the 2nd and 3rd year of their PhD studies. These monitoring measures ensure that the students are able to submit a thesis of the required standard within the timeframe.<br>*Creative input of PhD candidates:* The continuous interaction of the PhD candidates with their supervisors, the Dissertation Committee and the whole faculty provide ample opportunity for creative input.<br>*Qualifications of*	

				consisting of three researchers with adequate knowledge of the subject, and who are independent from the project and have obtained a doctoral degree. A written report describing progress in learning and science is submitted and the student gives an open research seminar. (2017: A 10-page literature review is now also required). The board, together with the supervisors and the doctoral student, shall assess the prospects of the project leading to a doctoral degree, and propose any necessary changes to the individual study plan. In the case of insufficient quality at this point it is the duty of the review panel to clearly indicate this to the Departmental Director of Doctoral Education, who in turn must meet with student and supervisor to discuss a plan of action for improvement.<br>It is inherent in the Governmental Higher Education Ordinance that students should progress towards being independent scientists by providing input.	*supervisors:* Supervisors in a thematic PhD program (faculty) are qualified university teachers (with a venia legendi certification, i.e. "habilitation" following a peer-reviewed evaluation of their research and teaching excellence), who are scientifically active within the respective field. The Dean of Doctoral Studies appoints the members of a program based on the recommendation of the spokesperson of the program. This appointment is granted only on the basis of an excellent track record in publication and external funding as well as experience in student supervision. See also: http://www.medunigraz.at/fileadmin/studieren/phd/pdf/VStudienplan_PhD_v8_ENGL.pdf	
9	*A PhD programme should only be initiated when the resources for completion are available. (BS3.4)*	Students are not allowed to enter laboratories that cannot demonstrate adequate funding. This is	The direct costs of research are funded by grants available to the supervisor. Supervisors must have a	As a Swedish PhD encompasses a 4-year period and as most research grants usually	Thematic PhD programs are established following submission of a proposal to the Dean of Doctoral	1

		assessed at least twice yearly by the BRET office and department chairs.	stable, externally-funded research program. The supervisor must commit to funding the student stipend, though every effort will be made to obtain studentship funding.<br>These stipulations are consistent within the College of Medicine, though not common to FGS, and will be found in Departmental Supplementary Regulations. See, for example, the document for my home department, Physiology and Pathophysiology http://umanitoba.ca/faculties/health_sciences/medicine/units/physiology/media/Survival_Guide_2015.pdf	encompass 2- or 3-year periods, then the economical, human and material resources are assessed at admission application by the Departmental Admissions Board and the Departmental Administration as a risk assessment. Based on the track record of the supervisor and the perceived possibility of maintaining resources required for the whole of the doctoral education period, the Department makes a decision to admit a student or not. In the event that resources should become limiting for some unexpected cause, the Department has responsibility for providing additional economic, human and material resources as required in order that a student can complete their studies within the 4-year period.	Studies. The Dean invites international experts to evaluate the proposal according to the following criteria: (i) Scientific quality of the proposal, (ii) accordance with the strategy of the university, (iii) future potential of the program, (iv) international and national networks, (v) existing potential (faculty, resources, previous achievements), (vi) critical size of the faculty, (vii) proof of compliance with the prerequisites for supervision and integration of PhD students in productive working groups, (viii) appropriate funding of the program via external grants (see also: http://www.medunigraz.at/fileadmin/studieren/phd/pdf/VStudienplan_PhD_v8_ENGL.pdf). An evaluation board comprising the Dean of Doctoral Studies, the Study Rector, the Vice Rector of Studies and Teaching, and the Speaker of the Academic Board for Doctoral Studies decide on the final approval of a program.<br>The majority of PhD positions at MUG are funded by external peer-reviewed grants. In addition, a total of 10 PhD candidates' positions are funded by MUG each year. These positions are	

					allocated to faculty members who have also been able to obtain external funding for PhD positions. PhD projects are started only if funds to cover the salary of the PhD candidates and the lab expenses are secured for a full 3-year period.	
4. PhD training programme						
10	*Programmes should be based on original research, courses and other activities which include analytical and critical thinking. (BS4.1)*	Each program provides supporting course work, journal clubs, presentation opportunities, and support with a mentored, critically evaluated research experience. The link in the adjoining box provides access to the description of each program.	The Academic Guide of the Faculty of Graduate Studies is comprehensive regarding all elements within this standard. Please refer to Section 5 of this guide: http://crscalprod1.cc.umanitoba.ca/Catalog/ViewCatalog.aspx?pageid=viewcatalog&catalogid=280&chapterid=3156&loaduseredits=False http://crscalprod1.cc.umanitoba.ca/Catalog/ViewCatalog.aspx?pageid=viewcatalog&catalogid=280&chapterid=3156&topicgroupid=18033&loaduseredits=False	Each individual study plan is meticulously prepared for each PhD student. Apart from details about specific input from all supervisors, time and economic plans, there is a suggested project plan for a 4-year period with described personalised ILOs, as well as definition of all taught courses, journal/book club, conferences and study visits that encompass the different learning activities that are expected to allow for development of a critical scientific approach.	The PhD program is structured in interdisciplinary thematic programs. The objective of thematic PhD programs is to provide students with a state-of-the-art research training in a particular field of biomedical science. The transfer of expertise includes the theoretical, methodological and experimental foundations of scientific research and the ability to critically analyse and evaluate research advances. The achievement of these goals and the ongoing development of the curriculum are continually monitored and discussed in the regular meetings of the Academic Board for Doctoral Studies together with the candidate representatives as members of the board. MUG offers the opportunity for PhD training in the following state-of-the art programs: PhD Program in Molecular Medicine (MolMed), Doctoral College	1

					in Metabolic and Cardiovascular Disease (DK-MCD), Doctoral College in Molecular Fundamentals of Inflammation (DK-MOLIN).	
11	*Programmes should be performed under supervision. (BS4.2)*	Supervisors (mentors) are selected by each Ph.D. granting department or program. Selection is based on experience, funding, and resources. Junior faculty are assigned a team of senior faculty who mentor the junior faculty member.	Please see Section 5.2 regarding the Advisor, Co-supervisor and Advisory Committee http://crscalprod1.cc.umanitoba.ca/Catalog/ViewCatalog.aspx?pageid=viewcatalog&catalogid=280&chapterid=3156&topicgroupid=18033&loaduseredits=False	At least two supervisors are appointed for each PhD student<br>at least one of the supervisors must be an Associate Professor or a professor<br>the principal supervisor (under normal circumstances) must be active at KI<br>at least one of the supervisors, preferably the principal supervisor, must be active in the department to which the doctoral student is admitted.<br>A doctoral degree is required for a person to be appointed as supervisor.<br>All supervisors shall be thoroughly familiar with KI's regulations governing doctoral education, and a mandatory supervisor training course must be conducted before becoming a principal supervisor for all new supervisors.<br>A web-based course must be conducted by all senior supervisors, as well as supervisors from other Universities.<br>In addition, other PhD students, postdocs or	The PhD program is structured in interdisciplinary thematic programs. Members of a thematic program (faculty) are qualified university teachers (with a venia legendi certification, i.e. "habilitation" following a peer-reviewed evaluation of their research and teaching excellence), who are scientifically active within the respective field and act as supervisors of PhD dissertations. University teachers from other universities may also become members of a program and act as supervisors. The supervisor chairs the Dissertation Committee which includes at least two co-supervisors. See also: http://www.medunigraz.at/fileadmin/studieren/phd/pdf/VStudienplan_PhD_v8_ENGL.pdf	1

				technical staff within a specific research group may give the PhD student daily support.		
12	*Programmes should ensure that PhD candidates have substantial training in ethics and responsible conduct of research. (BS4.3)*	All entering PhD students and postdoctoral fellows take Responsible Conduct in Research courses. This is supplemented by seminars throughout training and a formal refresher every 4 years.<br><br>http://research.vanderbilt.edu/rcr/	All PhD students are required to take the course on Academic Integrity. http://umanitoba.ca/faculties/graduate_studies/registration/grad7500FAQ.html The Institution has a strong policy framework on Research Integrity and Responsible Conduct of Research and this applies to all researchers, including students. http://umanitoba.ca/researchintegrity/ Presentations on this topic are high-profile in the institution and students are expected to be aware/held accountable in this matter. As most of these presentations are not within a course format, we do not have exact numbers, but I expect that all students have been exposed to these requirements and resources. Enrolment in iMED 7140, a specific course on these topics is typically 20.	There is a formal requirement for attendance of an research ethics course as part of the Basic Course (ECTS) requirement, encompassing both research ethics concerning humans and animals as well as the ethics of correct research conduct and publication practicies. In addition, PhD students are expected to participate in preparation of ethical permits associated with their PhD projects.	Scientific integrity and ethical issues in clinical trials, animal experimentation and related work are dealt with in the introductory seminar on "Fundamentals for graduates in medicine and dental medicine or science and engineering" as well as in "Fundamentals of scientific research and general skills".	1
13	*Programmes should have clear 3-4 year timeframe. Extensions should be possible but limited and exceptional. (BS4.4)*	Time in the US for a Ph.D in the biomedical sciences is about 5.6 years. Programs at Vanderbilt are not significantly different than this value.<br>Any student who enters year 7 of training must have approval after petitioning	Four years would be an aspirational goal. "Time-to-completion"(TTC) is a topic of strategic focus at our institution, with an emphasis on decreasing the duration. At our institution the average TTC in the Life sciences is about 5.7 years.	4-years equivalent full-time study (8 semesters of 100% activity) is expected. While a sufficient number of scientific papers could be produced within a shorter timeframe, the scientific maturity expected of graduating students is	According to the national regulations, PhD programs have a minimum duration of three years. On average the candidates need about 3 and a half years to complete their PhD. The candidates enter a temporary employment contract for	3

		the graduate school to explain the reasons for an extended timeline.	A detailed report on our treatment of this topic is found in https://umanitoba.ca/faculties/graduate_studies/media/Report_of_the_TTC_Task_Force.pdf<br>The process for requesting a time extension is decribed in http://umanitoba.ca/faculties/graduate_studies/registration/registration_faq.html	expected to be first developed after 3 years. Extensions are possible.	three years (the minimum duration of the degree program) with all social benefits mandated by law. The contract is for a 75% employment. This form of employment allows candidates enough time to attend necessary courses and work on their dissertation. If the candidates have been at least 6 months abroad, MUG may prolong their contract for another year.	
14	*Programmes should include relevant courses totalling about 6 months. A substantial part should be concerned with training in transferable skills. (NB. "courses" can be liberally interpreted as scientific activities not directly related to the project). (BS4.5)*	All programs offer courses in scientific communication that may include an oral component, a written component, or both. Additional elective courses are available to all. The career development programs referenced above all have courses in communication and professionalism. The amount of time spent in these activities varies amongst trainees as they have the freedom to customize their participation. In most cases, this is a few hours a month.	Our institution does not approve courses not directly relevant to projects.<br>We do have a wealth of scientific activities not directly relevant to student projects, including teaching, English (both written and oral), and career development/alternate careers. These were described in BS 2.1 (above)	Corresponding to 30 ECTS are required for a PhD (20 weeks): Basic courses – corresponding to at least 7.5 ECTS<br>Subject-specific courses – corresponding to at least 9 ECTS<br>Conferences, teaching, study visits, journal clubs – corresponding to at least 4.5 ECTS<br><br>The remaining credits can be achieved through additional subject-specific or non subject-specific courses (e.g. about scientific innovation in the pharmaceutical industry, introduction to teaching), teaching activities, presentations at international conferences, research visits abroad) in order to satisfy the individual's future career requirements.	Candidates are trained according to rational scientific concepts and principles and are familiarized in both theory and practice with the methodology of scientific research. In journal clubs and thesis seminars, candidates are trained in verbal presentation, communication and critical evaluation of scientific issues. According to the curriculum, PhD candidates must present their current research findings at the MUG annual Doctoral Day or at an external (international) conference. The writing of annual progress reports and the preparation of laboratory protocols and reports for the completion of laboratory courses train candidates in written presentation and discussion, which prepares them for	2

				Generic skills are included in the basic courses, and these must be completed prior to the halftime review.<br><br>See the General syllabus: https://internwebben.ki.se/sites/default/files/allman_studieplan_eng_faststalld.pdf	writing the dissertation and the publication of the scientific results in international scientific journals.<br>In sum, PhD candidates are required to attend courses totaling about 6 months. Further information about the courses can be found at: http://www.medunigraz.at/fileadmin/studieren/phd/pdf/VStudienplan_PhD_v8_ENGL.pdf	
15	*There should be arrangements that allow PhD candidates to perform part of their programme in another institution, national and abroad. (BS4.6)*	This is considered on an individual basis. Some funds are available and many programs have formal exchange arrangements with local, national, and international partners.	We have numerous academic exchange programs and students may participate as per their interests or the availability of special populations, expertise or facilities elsewhere.	Study visits of variable time are recommended and frequently included in the individual study plan of many PhD students. These may be national or international. Specific international collaborative exchange PhD programmes currently exist with NIH and the Mayo Clinic (USA), Makerere University (Uganda) and in Singapore, as well as several EU-funded ITNs which allow interaction with both academic institutions and industrial institutions. Experience of more than a single research environment is thus highly recommended for all PhD students.	In principle, the curriculum makes it possible to complete mandatory courses in a flexible manner. The practical training takes place continually at MUG, and the coursework is scheduled such that the research activities are affected as little as possible. Although the curriculum does not explicitly provide for mandatory studies abroad, candidates are encouraged to complete a maximum of up to one semester abroad (with keeping their regular salary.)<br>A pivotal added value of the PhD program is the financial support for candidate mobility: This measure allows the candidates both to attend international meetings and workshops and, even more substantially, to carry out part of their thesis projects outside Austria.<br>MUG also offers the	1

					possibility to do a JOINT PhD.	
16	*Programmes that are performed in parallel with clinical/professional training should have the same time for research and course work as any other PhD candidate. (BS4.7)*	The MD, PhD and PharmD, PhD programs perform PhD research under the same guidelines and supervision as other PhD trainees.	We offer a MD/PhD Program whose PhD component is administered within the relevant departmental regulations/program committee. Its requirements, thus, are identical to those of the other PhD students in that discipline. The Dean for Undergraduate Medical Education sits on the MD/PhD Committee and ensures that adequate leave from medical studies is granted for these students.<br><br>http://umanitoba.ca/fckeditor/editor/faculties/health_sciences/medicine/research/grad_undergrad/6696.html	The requirements outlined in BS4.5 and all general requirements are the same for clinical and preclinical PhD students.<br>Many clinical students attend defined clinical 'Research Schools' in which the course activities are organised for a specific cohort of students, ensuring them approval of time away from their clinical duties.<br>A minimum of 50% defined time for doctoral education is a prerequisite for admission of a clinical PhD student, equating to 8 years accumulated time.	See also QD4.2. The PhD curriculum is designed to cover a 3-year period during which each PhD student is employed as a research associate. Typically, the employment contract covers 75% of the legally defined working hours per week to carry out the lab work. The 25% time not covered by the employment contract allows the PhD candidates to do their coursework and write the disssertation. As residency regulations require a 50% appointment for clinical duties at minimum, PhD training and residency cannot be pursued at the same time.	1
17	*Progress of PhD candidates should be continuously assessed by the institution throughout the PhD. (BS4.8)*	Students who enter IGP, QCB , or MSTP are monitored by those program directors prior to entering a Ph.D. granting program. Upon entry into any of the 10 degree granting programs, the Ph. D. program director is one point of oversight in addition to the mentor. Importantly, by year 2 in the degree granting program, the dissertation committee is formed and meeting regularly (no less than 9 months) with each trainee. The results of these meetings are provided for the program director.	At least annually, with a deadline provided by the FGS, the student and advisory committee meet to review progress and make recommendations.<br><br>http://umanitoba.ca/faculties/graduate_studies/media/Progress_Report_2015.pdf	There are multiple points of evaluation and quality control, as described in BS3.3.	According to the PhD curriculum, the Dissertation Committee supports and advises the student in the subject area at least once a year in a formal meeting. The PhD candidate must present her/his interim report at this meeting, and the Dissertation Committee will provide written comments on the progress of the work. An extraordinary meeting of the Dissertation Committee may be requested by the supervisor, a Dissertation Committee member, the student, or the Dean of Doctoral Studies.	2/3

5. Supervision						
18	*Each PhD candidate should have a principal supervisor and when relevant at least one co-supervisor to cover all aspects of the programme. (BS5.1)*	Ph.D. candidates generally have one although informal co-mentoring relationships are not uncommon. Formal co-mentoring is possible.	PhD candidates typically have one Supervisor, though as appropriate, the additional expertise of a co-supervisor may be approved.<br>Please see Section 5.2 regarding the Advisor, Co-supervisor and Advisory Committee http://crscalprod1.cc.umanitoba.ca/Catalog/ViewCatalog.aspx?pageid=viewcatalog&catalogid=280&chapterid=3156&topicgroupid=18033&loaduseredits=False	All students must have a principal supervisor and at least 1 co-supervisor, although more than 4 supervisors in total is generally not recommended.<br>All students must have an external mentor that is independent from the research project. See BS4.2.	For each dissertation, the Dean of Doctoral Studies assigns a Dissertation Committee consisting of at least three persons who are headed by the supervisor. One member must be from outside the institute or clinical department where the work will be performed.	1
19	*The number of PhD candidates per supervisor should be compatible with the supervisor's workload. (BS5.2)*	The number of trainees is monitored and approved by the Ph.D. training program director.	The number of PhD students is approved by the departmental graduate program committee. Relevant information would include the size/composition (post-docs, research associates) of the research team.	Assessment of the academic, social and leadership skills of supervisors, as well as their perceived availability to efficiently supervise, is made by the Departmental Director of Doctoral Education and the other members of the Admission Board at the time of admission application. It is the Head of Department who takes the final decision concerning admissions.<br>It is the duty of the Director to restrict (or prevent) the number of PhD students allowed to be supervised by a given supervisor.<br>In one Department (Clinical Neuroscience) all potential supervisors must be approved by the	On average, a supervisor takes care of 2-4 candidates.	1

				Department annually before they can even be considered. (2017: Mandatory for all Departments)		
20	*Supervisors should be scientifically qualified and active scholars in the field concerned. (BS5.3)*	Supervisors must have an appointment in the graduate school and be approved by the Ph.D. granting program. This requires mentoring experience (junior mentors are assigned senior mentors), productivity as measured by publications, and funding.	Supervisors must be members of the Faculty of Graduate Studies and approved by the Departmental Graduate Program Committee, as well as be active in the research field (funded, publishing) and experienced mentors.	The supervisory team (principal and co-supervisors) must encompass sufficient scientific knowledge within the whole subject of study for an individual PhD student. Different supervisors may thus contribute with different, complementary competences. Perceived scientific track record of scientific quality is inherent in review of supervisor suitability at admissions procedure.	For each dissertation, the Dean of Doctoral Studies assigns a Dissertation Committee consisting of at least three persons who are headed by the supervisor (see QD5.4). Supervisors need, in addition, be members of a PhD program faculty, a position that is granted only on the basis of an excellent track record in publication and external funding. The faculty of supervisors consists of a well-balanced mixture of established and young scientists; more than 25% of the participating scientists are female. Further requirements are also described in BS3.3	1
21	*Supervisors should have regular consultations with their candidates (annotation: varies during the programme but will normally mean several times per month). (BS5.4)*	Most programs suggest weekly meetings between the trainee and the supervisor or mentor.	In the College of Medicine, it is typical that Supervisors hold lab meetings weekly and with individual trainees as their research findings require. Given the close contact of students in a laboratory setting, these interactions are an ongoing (daily) on an informal basis. https://umanitoba.ca/faculties/graduate_studies/media/Roles_and_Responsibilities_2012.pdf	A detailed plan of student-supervisor interactions is included in the individual study plan and discussed with student, supervisors, Departmental Director of Doctoral Education and Admissions Board during the admissions seminar. Daily, weekly or monthly physical meetings can be complemented by email/Skype/telephone interactions depending on the geographical locations of students and supervisors.	Supervisors and candidates are in constant contact. The candidates usually work in an inspiring environment together with other PhD candidates and postdoctoral fellows and present their project results regularly at lab seminars and data clubs. The candidates present and critically analyse the progress of the scientific project work at least once a year in a written Progress Report and a subsequent discussion meeting with their individual Thesis	1

					Committee. This serves to train both the written and verbal communication skills of the candidates. On the other hand, they receive feedback on the progress of their projects and also learn to view their own work critically.	
22	*It should be ensured that training for all supervisors and potential supervisors is available. (BS5.5)*	Some training courses are available through the BRET office. https://medschool.vanderbilt.edu/bret/faculty-resources<br><br>All junior faculty have mentoring committees that support their career development, including mentoring, until the faculty member is promoted with tenure.<br><br>Individual courses or mentoring is also available through the university. http://healthandwellness.vanderbilt.edu/<br><br>Support is also available from the office of faculty affairs. https://medschool.vanderbilt.edu/faculty/	As per https://umanitoba.ca/faculties/graduate_studies/media/Roles_and_Responsibilities_2012.pdf<br>It is the responsibility of department/unit heads to provide mentorship training for all supervisors.<br>The Centre for the Advancement of Teaching and Learning also offers a formal (certificate) "Mentor Program". http://intranet.umanitoba.ca/academic_support/catl/tlcmentor.htm	A basic (1-week) course in supervisor training is mandatory for all new supervisors. A web-based course (focus on KI rules) is required for all 'established' supervisors as well as for supervisors from other universities.<br>Advanced courses (1-week) are optional for increasing skills in either pedagogical reflection or in leadership skills. (2017: Principal Investigator course)<br>A series of optional subject-specific lunchtime seminars are hosted each term for the purpose of 'refreshing' or 'deepening' of knowledge, and include subjects such as conflict management, research ethics and ILOs.	The Office for Doctoral Studies organizes each year a 1-2 day workshop for supervisors in which all issues relevant to a successful supervision of doctoral candidates are addressed. In addition, MUG offers several continuing education courses which are also covering issues related to supervision, such as scientific integrity, mentoring and career planning.	2
23	*The supervisor-candidate relationship is the key to a successful PhD programme. There should be mutual respect and shared responsibility. (BS5.6)*	Students select mentors during rotations of several weeks duration during the first year of graduate school. This provides for students to sample up to 4 mentors. IGP students are coached as to how to recognize mentoring skills and how to build a mentoring relationship.	Our college has developed a Student- Mentor MOU based upon the template of the AAMC https://www.aamc.org/download/49868/data/gradcompact.pdf<br>We also have an office of Human Rights and Conflict Management that can be a	This is a cornerstone to the KI philosophy, and something that is discussed during the admission seminar with students and supervisors, during the PhD student introduction course, and during supervisor training courses.	The primary task of a faculty member is to supervise the thesis project of her/his candidate(s). They get to know each other at the hearing before accepting the candidates to the PhD program. At the hearing the faculty presents themselves and the proposed thesis	1

		https://medschool.vanderbilt.edu/bret/impact	good resource in this area. http://umanitoba.ca/human_rights/	Supervisors and students are matched by mutual consent and interaction prior to admission. Supervisor-student interactions are monitored by the Departmental Director of Doctoral Education, and should any conflict arise the Director has the responsibility to work for a solution of the problem.<br>Following a completed halftime review, in which the halftime review committee has indicated that they perceive problems in the supervisor-student relationship, then the Departmental Director of Doctoral Education must investigate and work towards a resolution of the problem.	projects; candidates are offered visits to the laboratories of their interest. Applicants are requested to nominate the supervisor and thesis project of their preference. The candidates' preference for the open PhD projects is then unblinded and matched with the preferred candidates of the supervisors.<br>In case of a personal conflict the candidate can call a mentor. The mentor, who is not necessarily a member of the faculty and definitely not a member of the department of the supervisor, advises the candidate how problems and misunderstandings with the supervisor might be resolved. If personal problems between supervisor and candidate persist, the mentor and the thesis committee may ask another faculty member or the Dean of Doctoral Studies to further supervise the PhD candidate, taking into consideration the candidate's preference.	
6. PhD thesis						
24	*The PhD thesis should be the basis for evaluating if the PhD candidate has acquired independent research skills and can evaluate work done by others. (BS6.1)*	The successful execution of a research program and the completion of a thesis is required for the Ph.D. in all programs.	The successful completion of a research program and the publication of a thesis is a core requirement for all of our Ph.D. programs.	This is inherent in the general and individual ILOs specified for each PhD student. The annual report for each student includes a self-reflection about perceived progress to this goal, and the halftime	Yes.	1

				review enables a formal assessment of progress towards this end.		
25	*The benchmark in health sciences is equivalent of three in extenso papers in scientific peer-reviewed international journals. If papers are in top-rank journals, fewer are acceptable. Manuscripts are also acceptable. It is the task of the assessment committee to determine if the material demonstrates 3-4 years of research at international level. (BS6.2)*	Content varies based on the discipline. A "body of work" agreed upon by the dissertation committee is the goal. All trainees must have one first author paper accepted prior to defending. The average number of papers across these programs is ~4. Obviously, some may have a single, high impact paper while others may publish several papers.	Given the breadth of our programs (biomedical through population health), the standards and norms vary widely by field, type of publication and impact, as well as the extent of collaboration within the laboratory. Thesis examining committees are charged with assuring that the student's contribution to original research is substantial and in accordance with national/international standards. The external examiner is usually an internationally recognized expert and his/her observations are given priority attention, both regarding recommended revisions and awards. A pass requires approval of the external examiner; not more than one of the internal may disapprove.	Most theses from KI are compilation theses, based on a number of separate original papers (not reviews). One of the constituent papers can be a systematic survey article, for example a meta-analysis. The requirement for such an article is that is creates new knowledge.<br>At least half of the constituent papers that are included in the compilation thesis must have been accepted for publication in a peer-reviewed journal. (2017: 2/3 papers published)<br>The number of constituent papers in a compilation thesis varies, but they must have a scope and quality that in total, in the opinion of the Examination Board, is equivalent to four years of full-time doctoral education. The doctoral student's contribution to the constituent papers must be clearly identified.	The formal curriculum of PhD studies at MUG requests a minimum of three years for the completion of the PhD thesis as well as one publication (or acceptance for publication) of the results of the thesis as first author in a peer reviewed, SCI-listed scientific journal. Usually the students publish additional papers (up to 3) in which they may be first author or, more frequently, co-author. It is the common understanding of the faculty members that the thesis projects and the publication(s) must meet internationally accepted criteria of originality, novelty, innovation, excellence and scientific relevance.	2
26	*In addition to papers, the thesis should include a full literature review and full account of aims, method, results, discussion and conclusion. (BS6.3)*	The introduction serves as a full literature review while most contain a comprehensive summary and statement of future directions.	This standard describes our expectations well.	A KI thesis usually comprises of 3 sections:<br>An overview of the subject area, with the purpose of demonstrating both broad and specific knowledge of the subject area, with appropriate referencing.<br>A summary of the work	For details see the PhD program curriculum: http://www.medunigraz.at/fileadmin/studieren/phd/pdf/VStudienplan_PhD_v8_ENGL.pdf<br>With the dissertation, the student provides evidence of having acquired the	1

				conducted, including *Aims, Methods, Results* and *general Discussion* and *Conclusions.* This should not be copied from the constituent papers/manuscripts, but instead provide the student with the opportunity to contextualise their findings with respect to the presented literature review, and to reflect on their learning experiences. The published papers and manuscripts. See also: https://internwebben.ki.se/sites/default/files/riktlinjer_ramberattelse_eng_2012.pdf	knowledge and ability to successfully conduct scientific research on current issues in an autonomous manner. Therefore, the dissertation must be wholly made up of independent original work that the student has produced and written up on her/his own. The student shall confirm that the rules of good scientific practice, as set out by MUG, were observed during work for the dissertation and in the related publications. A cumulative dissertation, for example consisting of an introduction and one or several publications, is not acceptable. The dissertation must be written in English. An abstract of the dissertation must be submitted in English and German. Thus, in addition to the required coursework and the comprehensive oral examination, the dissertation is the most important performance criterion in the graduation process.	
27	*If the thesis is presented in other formats (e.g. as single monograph), the assessment committee should ensure equivalence to the above benchmark. (BS6.4)*	None.	Other formats are not used.	A doctoral thesis may also be presented in the form of a monograph thesis. Special rules of review then apply.	None.	1
28	*A PhD thesis in clinical medicine should meet the same standards as other*	Yes.	Yes.	There is no distinction between clinical, preclinical or other student	Yes.	1

	PhD theses. (BS6.5)			PhD thesis requirements – the same rules apply throughout.		
7. Assessment						
29	*Acceptance of a PhD thesis should include acceptance of both written thesis and a subsequent oral defence. (BS7.1)*	Thesis is submitted to the dissertation committee at least 10 days prior to the oral defense. It is assumed that the mentor has approved the written document. At the oral defense, the first hour is open to the public and all questions are welcomed. An additional 2 hours is schedule for a closed examination with the committee where the written document is discussed as well as the oral presentation. Approval is by consensus or vote. If a vote is taken, a quorum of 3 of the 5 members is required. A majority vote is required to pass.	The procedures are described fully in the academic guide, see pages 67 – 69: http://umanitoba.ca/student/records/media/2015-2016_Graduate_Academic_Calendar_Aug_4_15.pdf	Before the thesis is submitted for printing, the Examination Board shall conduct a preliminary review in which the thesis' constituent papers are reviewed, together with details of the individual contribution of the PhD student, record of their formal learning experiences and their perceived performance as summarised by the main supervisor.<br>If accepted, the student defends his/her thesis before a faculty opponent and an Examination Board comprising of 3 senior researchers<br>A public thesis defence typically has the following format:<br>A brief summary of the subject area is given by the faculty opponent (10-20 min).<br>The student gives a short research seminar to present their work (20-30 min).<br>The opponent and PhD student discuss the thesis work (1-2 hours).<br>The examination board and PhD student discuss the thesis work (30 min).<br>Questions from the audience are then	The final decision regarding the completion of the experimental work and submission of the written thesis is carried out jointly by the candidate, the supervisor, and the Dissertation Committee, on the grounds of the achieved project aims, the quality and coherence of the results obtained and the quality of the publication. The quality of the thesis and the public defence are assessed by officially designated committees comprising two external reviewers and examiners. The supervisor and the other members of the Dissertation Committee must not serve as reviewers of the dissertation or as examiners at the oral defence.	2

				permitted.		
30	*PhD degrees should be awarded by the institution on the recommendation of assessment committee who have evaluated the thesis and the oral defence. (BS7.2)*	Recommendation is made by the members of the dissertation committee with the assent of the program director.	Yes.<br>see pages 67 – 69: http://umanitoba.ca/student/records/media/2015-2016_Graduate_Academic_Calendar_Aug_4_15.pdf	The Examination Board shall assess the thesis and its public defence with regard to:<br>the respondent's presentation of the thesis at the public defence, including the discussion with the opponent, and the ability to answer questions and discuss the significance of the results within the field of research<br>the quality of the comprehensive summary<br>the scientific content of the constituent papers<br>the fulfilment of the learning objectives of the doctoral degree.<br>Against the background of this assessment, the Examination Board comes to a decision to award a grade of either pass or fail.<br>.	Yes. For details see the PhD program curriculum: http://www.medunigraz.at/fileadmin/studieren/phd/pdf/VStudienplan_PhD_v8_ENGL.pdf<br>The thesis and the completion of the PhD curriculum are evaluated in a two-step process: review of the thesis and assessment of the oral defence. The supervisor and the other members of the Dissertation Committee must not serve as reviewers of the dissertation or as examiners at the oral defence.	1
32	*The assessment committee should consist of established and active scientists without connection to the milieu where the PhD was performed and without conflict of interest. Min. two should be from another institution (BS7.3)*	Generally 4 faculty members from within the training program and 1 from outside are required on each committee. Members from outside the institution are allowed but not common.	The internal members are normally members of the advisory committee (minimum of three, one of whom must be from outside the unit). One external (from outside the U of Manitoba is appointed by FGS.<br>see page 67 : http://umanitoba.ca/student/records/media/2015-2016_Graduate_Academic_Calendar_Aug_4_15.pdf	All of the members of the Examination Board must be associate professors or professors.<br>All of the members must be experts in the field of the project. The combined expertise of the Examination Board shall cover the entire content of the thesis.<br>The members of the Examination Board must be independent of and unbiased in relation to the doctoral student, the supervisors and the	The completed dissertation is first assessed by two reviewers. Eligible as reviewers are scientists who hold a teaching qualification (venia legendi) or a comparable qualification within the field of the dissertation, if they are not subject to any kind of conflict of interest, and if they are affiliated to a university other than MUG. The supervisor and the other members of the Dissertation Committee must not serve as reviewers	3

				project.<br>If possible, at least one member of the Examination Board shall have been a member of the board at the doctoral student's half-time review. Only one of the members may belong to the same department as the doctoral student (or the principal supervisor, in cases when the principal supervisor is in a different department as the doctoral student).<br>At least one member shall come from another university.	of the dissertation.<br>Following approval of the thesis, the doctoral program is completed with the final public comprehensive oral examination before a committee of three examiners . Eligible as examiners are scientists who do not have any kind of conflict of interest. Two of the three examiners must be affiliated with a university other than MUG.<br>The supervisor and the other members of the Dissertation Committee must not serve as examiners.	
33	*The supervisor should not be a member of the assessment committee (BS7.4)*	The mentors a member of the dissertation committee.	No. As per the Academic Guide of the University (p. 67), the Supervisor is explicitly a voting member of the Examining Committee. All members' votes are equal – supervisor does not have more weight than others.<br>http://umanitoba.ca/student/records/media/2015-2016_Graduate_Academic_Calendar_Aug_4_15.pdf	None of the supervisors take part in any of the formal assessment of the PhD. From 2013 it is recommended that the main supervisor *not* act as chairperson during the thesis defence, as has been the previous KI tradition.	For details see the PhD program curriculum: http://www.medunigraz.at/fileadmin/studieren/phd/pdf/VStudienplan_PhD_v8_ENGL.pdf<br>The supervisor and members of the Dissertation Committee must not serve as reviewers or examiners of the dissertation.	3
34	*If the assessment of the thesis/defence is negative, the PhD candidate should normally be given an opportunity to rewrite/an additional defence. (BS7.5)*	The student is given a single opportunity to correct any deficiencies.	The student is required to withdraw if he/she fails the thesis/defence twice. (See page 69 in the Guide)<br>http://umanitoba.ca/student/records/media/2015-2016_Graduate_Academic_Calendar_Aug_4_15.pdf	The Examination Board must provide a written justification when awarding a fail. The doctoral student will then have the opportunity to, at a later date, re-apply for a public defence of their thesis. However, there is no obligation on the part of supervisors, the	The final comprehensive oral examination is only deemed to be completed successfully if each examination subject has been awarded a grade of at least "satisfactory". If a grade "unsatisfactory" is given for more than one examination subject, the final comprehensive oral	1

				department or KI, to cover the additional costs that result from a renewed defence of thesis, or to support the doctoral student after a failed doctoral examination beyond the expiry of the existing appointment.	examination must be repeated entirely.	
8. Structure						
35	*The graduate school should have sufficient resources for proper conduct of PhD programmes. This includes resources to: Support admission of PhD candidates, implement the PhD programmes of the PhD candidates enrolled, assess PhD theses and to award PhD degrees. (BS8.1)*	Both BRET and the graduate school support the programs.<br><br>https://medschool.vanderbilt.edu/bret/<br>http://gradschool.vanderbilt.edu/	Resources of both the College of Medicine and the Faculty of Graduate Studies are available in support of graduate programs. The Dean of Medicine supports travel expenses for in-person attendance of external examiners at the thesis defence, a travel fund for graduate students to attend conferences and the Canadian Student Health Research Forum (based in Winnipeg) to network our trainees with the best nationally.<br>http://umanitoba.ca/graduate_studies/<br>http://umanitoba.ca/medicine/research/grad_undergrad/<br>http://umanitoba.ca/faculties/health_sciences/medicine/research/grad_undergrad/Assistant_Dean.html<br>http://umanitoba.ca/medicine/research_days/	The KI Board of Doctoral Education has overall responsibility for doctoral education within KI, and this is delegated to each Departmental Head.<br>The Board provides departmental funding for appointment of both Study Administrator and Director of Doctoral Education.<br>The Board also economically supports a Central Director of Doctoral Education, as well as several administrative officers responsible for the formal administration of doctoral education within KI, and finances the thematic Doctoral Education Programmes that are responsible for running diverse educational activities including all formal teaching courses required for PhD students.<br>In addition, the Board has responsibility for continual development of Doctoral Education with respect to formal practices, quality	*PhD candidate positions*: The description of and access to the facilities are given under BS1.2.<br>Every PhD candidate's position is funded with a salary of € 37.000 per year and a bench fee of € 10.000 per year.<br>*Administration*: The PhD program is supported by MUG with a full academic position (assistant to the Dean).<br>*Other expenses*: The budget for office, administration, travelling, hearings, guest lectures, summer schools and workshops is fully covered by MUG.	1

				assurance and follow-up.		
Quality Development						
1. Research environment						
36	*Institutions lacking facilities/expertise should collaborate with stronger institutions to reach required standards. (QD1.1)*	Vanderbilt has local, national, and international partners.<br><br>Locally we partner with undergraduate institutions such as Fisk and Tennessee State University and Meharry Medical School. These institutions serve underserved populations. We collaborate with Lipscomb College of Pharmacy for several programs.<br><br>National and International partners are too numerous to mention here.<br><br>Some examples are: http://as.vanderbilt.edu/overview/faculty/research/international/core.php<br><br>Many programs have additional collaborations. (ie pharmacology has a partnership with the Univ of Bonn)	Our programs are approved on the basis of ability to provide adequate resources (facilities, expertise).<br>In addition, we have numerous collaborative exchange agreements with other institutions to complement our existing programs and provide an enhanced educational experience.	Some forms of Intra-- or Inter--Departmental, Inter--Campus or Inter--University (National or International) collaborations are typical of a KI PhD project.<br>It is expected that the required standards are already fulfilled by the host laboratory without the need of collaborative support.	MUG provides a cutting-edge research environment. In exceptional cases and if needed, supervisors have collaborative arrangements for performing parts of the PhD projects at other institutions (for instance, at the Institute of Molecular Biosciences of the University of Graz).	1
37	*When relevant, PhD programmes should include time in another laboratory, preferably abroad. (QD1.2)*	Students often spend time in other Vanderbilt laboratories. Indeed, most if not all rotate through several laboratories. Students have funds if they wish to visit a laboratory for addition experience.	While there are numerous opportunities for participation at other institutions, these are not normally required.	It is highly recommended that students spend some period of time visiting an international research group with the purpose of extending their knowledge and experiencing a different research environment.<br>Such activities are	Almost all PhD projects are carried out in collaborations of the supervisors in different departments of MUG. According to the PhD curriculum, one of the co-supervisors of a PhD student must be affiliated with a department other than the one where the PhD project	1

				common especially among preclinical PhD students. A maximum of 6 higher education credits is given for a period of research with a research group at a different university.	is carried out. Stays abroad (up to 6 months) are actively encouraged by MUG, and about 20-25% of the candidates spend sufficient time abroad.	
38	*Possibilities for joint and double degrees should be explored. (QD1.3)*	Yes, MD, PhD. https://medschool.vanderbilt.edu/mstp/<br><br>Smaller PharmD, PhD program is directed by Pharmacology	We offer an MD/PhD program for eligible students (documented above).	A designated International Relations office at KI central administration explores these types of interactions, and several such exchange programmes are already established (e.g. with Makere University in Uganda).	A PhD can be obtained within a Joint PhD Degree program. Candidates have to spend at least one year at the partner university working on their thesis to complete a Joint PhD. It is required that candidates submit an individual Joint PhD agreement to the Dean of Doctoral studies for approval. Within the Joint PhD agreement, all work done and exams passed at the partner university are recognized.	1
3. Admission policy and criteria						
39	*In choosing PhD candidates, the applicants potential for research should be considered, not just past academic performance. (QD3.1)*	Application links are found here: https://medschool.vanderbilt.edu/igp/<br><br>A great deal of emphasis is placed on research experience. Our metrics show that prior research success is the only predictor of success in graduate school.<br>All applications are reviewed and offers of interview given to those judged to combine research skills with academic accomplishment.	Students are selected for PhD programs based both upon achieving a minimum grade point average (required by FGS) and the research aptitude demonstrated in their Masters program (research productivity, recommendation of the Masters supervisor and critical thinking demonstrated.)	Selection of PhD students is delegated to the supervisors that will be involved in the research project. Criteria for selection are therefore decided upon by the individual. See Rules for Doctoral Education Section 2.3:<br><br>Selection from amongst the applicants will take place on the following grounds:<br><br>documented knowledge of the subject that is of significance to the research area	Generally, applicants should have spent at least six months at the bench in a research laboratory during their undergraduate/master's studies. Calls for application are usually open for 8 weeks. Applicants are requested to submit an outline of their scientific background, research interests and motivation to join the program, along with the names of at least two former supervisors/teachers as reference. The letter of motivation is crucial in determining the extent of commitment to and the	1

				analytical expertise<br><br>other documented knowledge/experience which may be of significance for doctoral education in the subject.<br><br>A combined assessment of the applicants' qualifications and suitability will be conducted.	interest in biomedical research of the applicants. Successful applicants must have the following attributes, listed by priority:<br>• Profound and persuasive letter of motivation<br>• Excellent references from previous supervisors with respect to personal, curricular and scientific achievements<br>• Documented scientific experience, such as relevant diploma thesis, publications and/or congress presentations<br>• International study or research experience<br>• Good proficiency of the English language<br>• Reasonably fast undergraduate study and excellent grades	
40	*Projects should be externally assessed by written project description or presentation to panel of independent scientists. (QD3.2)*	Several students seek individual funding for projects. Only these are externally reviewed.	PhD projects are assessed in a research proposal to their advisory committee and other interested faculty members (this event is advertised to the larger college community).	The Admissions Board within each Department is appointed by the Department and includes the Departmental Director of Doctoral Education, at least one student representative and at least 3 senior scientists with subject-specific	Most PhD projects and positions are funded by peer-reviewed grants. Hence they have been subjected to competitive assessment by external reviewers. PhD projects funded directly by MUG are not externally assessed but given only to faculty	2

				competences to cover areas of study within the Department (large Departments have larger Admissions Boards).<br>In case of lack of specific subject-specific competence within a Department then appropriately competent scientists from other Departments (or externally of KI) are identified and asked to review the research plan.	members with an excellent track record in publication and external funding.	
41	*PhD candidates should have rights and duties commensurate with the value (to the institution) of the research performed. (QD3.3)*	All candidates are paid US$28,500 per year with full tuition remission and health insurance.	All PhD students receive a stipend of at least $18,000. In addition, we have a top-up (incentive) program of $6000 for students who compete successfully for national studentships.	This is in accordance with KI philosophy.	PhD candidates have free access to all lectures, seminars, courses and workshops associated with the PhD curriculum, and to experimental techniques, facilities and equipment in the laboratories of faculty members. Candidates are requested to perform the work of their thesis independently and at high international standards as exemplified by publications in SCI-listed scientific journals. . Candidates The PhD candidates enter a temporary employment contract for three years (the minimum duration of the degree program) with all the social benefits mandated by law.	1
42	*If the PhD candidate is obliged to obtain extra income, it should be ensured that the PhD candidate has the necessary time to*	Students are strongly discouraged from working. All are given full tuition remission, health insurance, and a stipend of US$28,500 per year.	The PhD program is considered to be a full-time engagement and it is not expected that students will be taking on other paid duties.	Parallel activities can be defined up to a maximum activity of 50%, but the individual study plan timeline reflects only time spent during doctoral	See QD3.3	1

	complete the program. (QD3.4)e			education and has to reflect the requirement of 4-years full-time equivalent time spent with the doctoral studies.		
4. PhD training programme						
43	*Merit should be given for courses taken elsewhere or other relevant experience. (QD4.1)*	Graduate level courses from other institutions may be accepted for transfer. Of the 72 hours required for a Ph.D., 24 must be in residence.	FGS vets previous academic credentials and credit may be granted as appropriate. Se section 5.4.3 in http://crscalprod1.cc.umanitoba.ca/Catalog/ViewCatalog.aspx?pageid=viewcatalog&catalogid=280&chapterid=3156&topicgroupid=18033&loaduseredits=False	Any educational activity or experience that is relevant for the individual study plan for the PhD student can be approved by the Departmental Director of Doctoral Education for accreditation.	Merit may be given for courses which have been attended at other doctoral programs or at other institutions according to the curriculum.	1
44	*For PhDs performed by clinicians, leave-of-absence from clinical duties should be provided for the PhD part of such programmes. (QD4.2)*	This is the norm for M.D., Ph.D. students.	The Associate Dean for Undergraduate Medical Education grants leave from medical studies for the PhD portion of the MD/PhD student's training.	Every clinical departmental head has to sign the individual study plan for each clinical PhD student, guaranteeing the minimum leave of absence required for enrolment in a PhD programme, which is 50%. Clinical PhD students are recommended to apply to the various clinical research schools in which cohorts of students receive the educational activities together at defined times, in which case their participation is guaranteed by the host clinic.	The PhD curriculum is designed to cover a 3-year period. Each PhD student is employed as a research associate for 3 years. Typically, the employment contract covers 75% of the legally defined working hours per week to carry out the lab work, and the salary is equivalent to that of a young scientist with a master's degree. The 25% time not covered by the employment contract allows the PhD candidates to do their coursework and write the dissertation. As residency regulations require a 50% appointment for clinical duties at minimum, PhD training and residency cannot be pursued at the same time.	1
45	*The graduate school should offer confidential*	I fully equipped counselling center, student health	*Independent counselling is available for hte range of*	Every student has the following options for	In case of a (personal) conflict there are several	1

	PhD candidate counselling concerning the PhD programme, supervision, personal matters etc. (QD4.3)	center, and a learning center are available to students. https://medschool.vanderbilt.edu/pcc/ https://medschool.vanderbilt.edu/student-health/ http://cft.vanderbilt.edu/	*student needs (academic, emotional, financial, etc). http://umanitoba.ca/student/counselling/ http://umanitoba.ca/student/advocacy/*<br><br>*As these services are confidential, we are not informed as to numbers.*	formal counselling: Departmental Director of Education: First-line support. Departmental Head: Preferred choice in case of involvement of the Departmental Director of Doctoral Education in the situation. Central Director of Doctoral Education: Preferred choice in case of involvement of Dept Head or Dept Director in the situation. External Mentor: Defined at admission as a trustworthy confidant. Doctoral Ombudsman: Employed by the Student Union (including both undergraduate and graduate students) to mediate and support all PhD students (confidentiality). Dean of Doctoral Education: has the ultimate responsibility delegated by the KI President, and can be directly involved in the process if the student wishes. Healthcare professionals: In case of the need for counselling this service is free for students (confidentiality).	options of confidential counselling for PhD candidates:<br>1) PhD candidate speakers: The candidate speakers officially represent the PhD candidates within the Faculty, towards the Executive Board of the PhD program, the Dean of Doctoral Studies and in public.<br>2) Representatives of the Austrian Student's Union: The Austrian Students' Union (ÖH) is the general representative body for all students in Austria. Every student in Austria is automatically a member of the ÖH. There is one ÖH at every Austrian university, who acts on behalf of the students at the respective university.<br>3) Office for Doctoral Studies: Whenever needed, the Dean and Vice-Dean of Doctoral Studies offer consultation in a trustful atmosphere.<br>4) Personal mentors: Each PhD candidate can choose a mentor who may not be a member of the faculty. The mentor provides additional advice on the candidate's career plans and supports the candidate to cope with professional and personal conflicts related to work.	
46	*Graduate schools should*	Committees are required for	All PhD students have a	It is recommended that all	On enrolment of a PhD	3

	consider having a Thesis Committee for each PhD candidate that monitors the progress of the PhD candidate through meetings with the PhD candidate and the supervisors. (QD4.4)	all trainees and are usually appointed in year 2 of training. Committees are composed of 5 members, 4 within dept, one outside. Committees are formed cooperatively by students and mentors. Each must be approved by the Ph.D. program director and the graduate school.	thesis advisory committee of faculty members who have expertise or experience considered useful to the student's program. See section 5.2.3 in http://crscalprod1.cc.umanitoba.ca/Catalog/ViewCatalog.aspx?pageid=viewcatalog&catalogid=280&chapterid=3156&topicgroupid=18033&loaduseredits=False	three or some of the halftime review committee be included in the final thesis defence examination committee, in order to contextualise better the progress of a give student. Each student, in addition to 2 or more supervisors, has an external mentor as an additional confidant/advisor during the duration of their studies that allows for social monitoring and evaluation of career planning, in addition to that conducted by the supervisor team. Appointment of a defined thesis committee has been discussed, but is considered to be both unnecessary given the current structure, and unmanageable considering the volume of PhD students within KI.	student, a Dissertation Committee is set up, consisting of the supervisor and 2-3 co-supervisors, one of whom need be affiliated with a department other than that where the thesis project is carried out. It is the central responsibility of the Dissertation Committee to co-supervise the progress of the PhD candidate's research throughout the PhD project as well as the curricular activities of the candidate. Members of the Dissertation Chaired by the supervisor, the Committee meets at least once a year for detailed discussion following a formal presentation of the progress of the project by the candidate.	
47	*The graduate school should encourage and facilitate PhD candidate involvement, and interact with PhD candidate representatives regarding design, management and evaluation of PhD programmes. (QD4.5)*	Each Ph.D. program has a student organization with representation on the graduate student council. Any student may contact the Dean of the Graduate School or the Senior Associate Dean for Research in the School of Medicine.	Representatives of the Graduate Students Association sit on the most prominent committees of the Faculty of Graduate Studies and on the College of Medicine Committee for Graduate Studies.	Student representatives are included in all KI Boards (Education, Doctoral Education & Research), in Departmental Steering Boards, in Departmental Admission Boards, and in sub-groups or other under organisations of these (e.g. dissertation committee, recruitment committee). Student influence is thus encouraged at every level and is taken seriously.	The students in each PhD program elect two PhD candidate representatives who officially represent the PhD candidates within the faculty, toward the Executive Board of the PhD program, the Dean of Doctoral Studies and in public. The PhD candidate speakers organize meetings of the PhD candidates' assembly, report to the faculty and the Executive Board and assist the	1

					Executive Board in the planning of courses and examinations, evaluating the curriculum and providing feedback of any kind.	
48	*There should be an appeal mechanism allowing PhD candidates to dispute decisions concerning their programmes and thesis assessment. (QD4.6)*	Any student may appeal to the program director or Chair, contact the Senior Associate Dean for Research in the School of Medicine, or the Dean of the Graduate School. Appeals or accusations of misconduct are taken seriously and may also include the office of faculty affairs or the provost.	Appeals procedures are documented in: http://crscalprod1.cc.umanitoba.ca/Catalog/ViewCatalog.aspx?pageid=viewcatalog&catalogid=280&chapterid=3156&topicgroupid=18028&loaduseredits=False	In accordance with the Governmental Higher Education Ordinance the following decisions can be disputed by PhD students: Accreditation of ECTS-denoted activities Formal mandatory activities with PhD Withdrawal of support by the Department Denial of obtaining examination certificate Non-approval of a thesis defence cannot be disputed but re-examination at a later date can be arranged in agreement with all parties.	There are several possibilities for PhD candidates to dispute decisions concerning programs and thesis assessment:<br>1) PhD candidate speakers: see QD4.3<br>2) Austrian Students' Union: see QD4.3<br>3) Dean and Office for Doctoral Studies.	1
5. Supervision						
49	*Responsibilities of each supervisor should be explicit. (QD5.1)*	Program directors generally provide orientation for new mentors. Mentors are also reminded of responsibilities by other members of the dissertation committee.<br><br>Often responsibilities are enumerated by each program. Example in Pharmacology is:<br><br>https://medschool.vanderbilt.edu/pharmacology/files/pharmacology/images/Mentor%20Handbook%20-%20Current.pdf	Within the College of Medicine we have developed (in consultation with the Graduate Students Association), a mentorship document: https://umanitoba.ca/faculties/health_sciences/medicine/research/grad_undergrad/media/MOU.pdf The Academic Guide of the institution (previously referenced) stipulates the formal responsibilities of supervisors. The ethical responsibilities of mentors are documented in the "ROASS" document:	These are clearly defined in the Rules for Doctoral Education: The principal supervisor shall have the overall responsibility for supervision when it comes to the planning and execution of the research project. The principal supervisor also has, together with the doctoral student, a responsibility to ensure that the doctoral courses and other elements that are specified in the general syllabus and individual study plan are	The supervisors guide the PhD candidates, provide the required resources and materials, coordinate the cooperation of the candidates within the research group, determine the details of the education program (lectures, courses, trips abroad, etc.) together with the candidates, support and advise the candidates in the preparation and presentation of results (papers, conference papers, dissertation) and in all other matters related to the thesis	1

			http://umanitoba.ca/student/resource/student_advocacy/academicintegrity/Academic-Integrity-policies-and-procedures.html	completed. The principal supervisor shall work to ensure that the annual follow-up, as well as the half-time review and defence of thesis, or licentiate degree, are planned and implemented. The principal supervisor is responsible for ensuring that the funding plan in the individual study plan is drawn up and revised.<br>A co-supervisor is primarily responsible for providing complementary scientific expertise to the project. The role of the co-supervisor shall be clearly stated in the individual study plan.	project and degree program. The supervisors and candidates agree to comply with the Standards of Good Scientific Practice of MUG. The responsibility of the supervisor is formally agreed upon in the thesis agreement which is signed by the candidate, the supervisor, the co-supervisors, the head of the graduate school and the Dean of Doctoral Studies.	
50	*Supervisors should have broad local and international scientific networks. (QD5.2)*	Criteria of publication and funding must be met. It is unlikely faculty will meet these criteria if they are not connected and known in their discipline.	This criterion is met indirectly through the requirement that supervisors be active members of the broader research community – publishing, funded and recognized.	Where possible and as defined by the requirements of an approved individual study plan, the ability of a PhD student to benefit from the local, national and international networks of the supervisor(s) is recommended. Extensive networks might not be expected for more junior, first-time supervisors, and this will not prejudice acceptance of a supervisor.	Supervisors of PhD candidates are qualified by:<br>Excellence in research as evidenced by publications and funding records<br>Experience in supervising PhD candidates<br>Practice in lecturing<br>Interdisciplinary approach and commitment to the goals of the PhD program<br>Leadership role in research programs and academic activities<br>Supervisor candidates apply for membership in one of the PhD programs and are evaluated by the program faculty. Following recommendation by the faculty, supervisors are elected by the Dean of	1

					Doctoral Studies.	
51	*Supervisors should assist with career development. (QD5.3)*	Please see above response for career resources. Mentors are encouraged to aid in career development. Most do and discussions of this aspect at each committee meeting are document. Members of the committee, program directors, and dept chairs also may assist. Importantly, the recently developed ASPIRE Program is available to all students throughout their training.	As per our Mentorship Compact, the expectations regarding career development are explicit. https://umanitoba.ca/faculties/health_sciences/medicine/research/grad_undergrad/media/MOU.pdf	This is an explicit task given to appointed supervisors and instructions to this effect are included in the basic supervisor training course. The (at least) annual personal evaluation discussion staged by supervisor and student includes discussion of career development (see Personal Evaluation assessment form).	The Dissertation Committee (supervisor and co-supervisors) supports and advises the PhD candidate concerning his/her career. In addition, MUG has set up a Mentoring Program for Scientists which is also open to PhD candidates. In this program, experienced members of MUG mentor young scientists. In their mentoring relationship, mentors act as role models, examples and counsellors.	1
52	*Institutions should consider having contracts on the supervision process, signed by supervisor, PhD candidate and head of graduate school. (QD5.4)*	We use the Individual Development Plan.<br><br>https://medschool.vanderbilt.edu/bret/individual-development-plansidps	Our Mentorship Compact is the basis for our students' and supervisors' undertaking in the matter of reciprocal responsibilities. https://umanitoba.ca/faculties/health_sciences/medicine/research/grad_undergrad/media/MOU.pdf	The individual study plan serves as a formal contract for mutual understanding of acceptance of the roles of students, supervisors and Departmental Heads. In addition, some Departments have additional MOU documents that are countersigned by the Director of Doctoral Education, student and supervisors.	On enrolment of a PhD student, a dissertation agreement (contract) is set up and signed by the candidate, the supervisor, the co-supervisors, the head of the graduate school and the Dean of Doctoral Studies.	1
53	*The principal supervisor, at least, should have some formal training as supervisor. (QD5.5)*	This is not uniform across mentors.	This aspect is not a common feature in our educational system. FGS offers workshops on mentorship, but not courses on supervision. In completing the various required (by FGS) elements of supervision and meetings of the advisory committee, it could be considered that, in combination with mentorship, the objective is	This is a prerequisite for all supervisors, as detailed in BS5.5. To date 1467 have completed the basic training course and 227 have completed the advanced courses.	All of the supervisors have passed a career evaluation referred to as habilitation, which is a peer-reviewed process to assess their achievements in research and teaching.	3

			met.			
54	*Supervisors should where possible also act as co-supervisors for PhD candidates at other graduate schools. (QD5.6)*	Mentors may have students in more than one Ph.D. granting program.	This is not common, given the geographical distance between graduate schools in our country (the nearest, for us, is 700 km distant, at the University of Minnesota, USA).	We do not have a graduate school system, but supervisors are often involved in the co-supervision of PhD students between different KI Departments, between different KI campuses, and between Swedish Universities.	Supervisors frequently act as co-supervisors especially at the University of Graz and Graz University of Technology. The co-supervisors are often affiliated with institutions other than MUG.	1
6. PhD thesis						
55	*The thesis should be written and optimally also defended in English, unless national regulations stipulate otherwise. An abstract of the thesis should be published in English. (QD6.1)*	All in English.	The language of our College is English throughout and all theses, abstracts and publications are in English.	The thesis is recommended to be written in English. Translation of the scientific *Abstract* to Swedish is an option that some Swedish students adopt. The thesis defence is almost always defended using English, and this is the KI recommendation. In the case of an 'all-Swedish faculty opponent/ committee' and at the expressed wish of either opponent or PhD student, the Swedish language is sometimes (rarely) used (most typically by some clinical students).	The PhD program is conducted exclusively in English. The dissertation must be written in English. An abstract of the dissertation must be submitted in English and German.	1
56	*If articles/manuscripts are joint publications, co-author statements should document that the PhD candidate has made a substantial and independent contribution. Ownership of results from PhD studies should be clearly stated. (QD6.2)*	The dissertation committee follows the student during data acquisition and analysis. The committee is aware of data ownership and issues of publication and authorship are addressed throughout the thesis work.	The matters of student contribution to multi-author work and team science within the context of the student's PhD program are dealt with by the Thesis Advisory Committee.	For each published article, submitted article or manuscript included in the PhD thesis, the contributions of the defending PhD student are explained as part of the PhD thesis defence application. In this paperwork it is clearly stated to what degree the	One paper (accepted), with the candidate as first author, is required for submission of a thesis for evaluation by two external reviewers. In the thesis, the candidate has to sign a declaration that she/he has made a substantial and independent contribution to the work and that any	

				defending student has contributed to the scientific concept, conduct, interpretation and publication of each article. It is also clearly indicated if the articles will be used in another PhD thesis. In accordance with Swedish law a PhD student owns all of their own results, so this is not an issue.	contribution from other parties is clearly identified.	
57	*PhD theses should be published on the graduate school's homepage, preferably in extenso. If patent or copyright legislation prevent this, at least abstracts of the theses should be publicly accessible. (QD6.3)*	Each are published electronically. Distribution may be limited for 1 year, after that time the documents are freely available.	All theses are published electronically through the University's library system.<br><br>The impact of patents pending on publication (up to 1 year) is dealt with through policy: http://crscalprod1.cc.umanitoba.ca/Catalog/ViewCatalog.aspx?pageid=viewcatalog&catalogid=260&chapterid=2896&topicgroupid=16553	An electronic PDF copy is made of each doctoral thesis and published at a defined KI thesis database website (http://publications.ki.se/xmlui/handle/10616/1?locale-attribute=en) The electronic version is not uploaded until the thesis defence has been approved by the KI Dissertation Committee.	The dissertations are published on the Internet in PDF/A format. In justified instances (e.g. legal patent aspects, non-disclosure notices for cooperation) a request to postpone publication for a maximum of 5 years may be submitted to the Study Rector.	1
58	*There should be a lay summary of the thesis in the local language. (QD6.4)*	No lay summary but a two page general abstract is written.	Lay summaries are not required, but the abstract, in English, is required.	While this is common for Swedish PhD students it is not a requirement, as English is the official language of Doctoral Education at KI.	An abstract of the dissertation must be submitted in English and German.	1
7. Assessment						
59	*The oral defence should be open to the public. (QD7.1)*	The oral defense is advertised across the entire campus and is open to the public.	The oral defence is broadly advertised within the University community and open to the public.	The PhD defence is open to the public. Only the subsequent deliberation of the 3 PhD members of the thesis committee and the faculty opponent are closed proceedings, during which the final decision of approval/non-approval is made.	The final comprehensive oral examination (PhD defence) is conducted as a public oral examination and open to the public.	1

				.		
60	*Where possible at least one member of the assessment committee should be from another country. (QD7.2)*	This is not routine.	The External Assessor is always from another Institution (at minimum, another province, with the nearest being 800 km distant).	A PhD defence comprises a detailed examination/discussion by an appointed faculty opponent, and further examination/discussion by an appointed panel of 3 experts within the scientific field. At least one member of the panel must arise externally from KI. It is more common in Sweden that an international guest assumes the role of faculty opponent.	The independent assessment committee for the PhD defence consists of three members, two of whom are external (one of them frequently from abroad) and one is a member of MUG. The supervisor and co-supervisors are not allowed to be part of the committee. Emphasis is put on the fact that there is no conflict of interest between candidate and any of the committee members.<br>.	3
61	*Apart from the thesis, the institution should ensure that sufficient transferable skills have been acquired during the PhD programme. (QD7.3)*	Most programs require formal courses in written and oral communication. All programs require participation in journal clubs and works in progress. All students can participate in leadership/transferable skills course and opportunities that are available across campus.	These aspects are reviewed within the Progress Review component of the Advisory Committee's deliberations.	The inclusion of transferrable skill training and evaluation of progression towards this end are inherent in the PhD programme described in the individual study plan, which is approved on admission and evaluated thereafter by the Department.	The training of the PhD candidates in their coursework and dissertation project provides them with the following general skills:<br>•Ability to work effectively in a team<br>•Ability to analyse information<br>•Ability to communicate effectively<br>•Ability to learn independently and to obtain information<br>•Awareness of ethical and professional responsibility<br>•Leadership skills	1
8. Structure						
62	*There should be procedures for regular review and updating of the structure, function and quality of PhD programmes, including both supervisor and*	Each graduate program is reviewed by the institution every 10 years. Many programs do more frequent reviews. Many of the programs are supported by government training grants	All graduate programs are subject to thorough review, with external assessors, every 7 years. This process is described in: http://umanitoba.ca/graduate_studies/admin/123.html	There are multiple points of evaluation and quality control, as described in BS3.3, including annual review and halftime review procedures.	All issues of the curriculum are regularly evaluated by the Academic Board for Doctoral Studies, set up by the Senate of MUG. The quality of courses, seminars and other curricular	1

	candidate feedback. (QD8.1)	which require a review and re-application at 3-5 year intervals.<br><br>Each student completes an exit interview at the end of the program.<br><br>Mentors are evaluated for inclusion on the list of mentors available to students on a twice a year basis.			activities are continuously evaluated by a structured feedback from the students. In addition, the PhD program has been subjected to an international accreditation process as well as evaluation by ORPHEUS. As a result, the PhD program was accredited by AQUIN, an independent agency, in 2010, and awarded the ORPHEUS label in 2015.	
63	*The graduate school should have a website in the national language and in English, including transparent information on policies concerning:*<br>*- background for the graduate school and the research environment*<br>*- expected outcomes of the PhD programmes*<br>*- description of the graduate school leadership and administration*<br>*- responsibilities of the head of the graduate school and the administration*<br>*- quality assurance and regular review to achieve quality improvement*<br>*- admission policy including a clear statement on the PhD candidate selection process*<br>*- the structure, duration*	https://medschool.vanderbilt.edu/bret/	Provide the web address for the graduate school. http://umanitoba.ca/graduate_studies/	An extensive body of information detailing all aspects is available at: http://ki.se/ki/jsp/polopoly.jsp?l=en&d=270 and at http://internwebben.ki.se/en/doctoral-education<br><br>Relevant documents are included as appendices (General Syllabus; Rules for Doctoral Education).	Web address for the graduate school: http://www.medunigraz.at/en/phd-medizin/<br>Web address of the Office for Doctoral Studies: http://www.medunigraz.at/themen-studieren/phd-medizin/office-for-doctoral-studies/	1

	and content of the PhD programme, including course requirements<br>*- type of courses offered, possible time in other institutions*<br>*- the methods used for assessing PhD candidates*<br>*- the requirements for the PhD thesis*<br>*- the procedure for assessing PhD theses*<br>*- the formal framework for following the progress of individual PhD candidates*<br>*- supervisor appointment policy, supervisor qualifications, duties of supervisors and duties of PhD candidates outlining the type, responsibilities and qualifications of supervisors*<br>*- effective use of information and communication technology*<br>*- appeal procedures. (QD8.2)*					
